# Specific SKN-1/Nrf Stress Responses to Perturbations in Translation Elongation and Proteasome Activity

**DOI:** 10.1371/journal.pgen.1002119

**Published:** 2011-06-09

**Authors:** Xuan Li, Olli Matilainen, Congyu Jin, Kira M. Glover-Cutter, Carina I. Holmberg, T. Keith Blackwell

**Affiliations:** 1 Joslin Diabetes Center, Harvard Stem Cell Institute, Harvard Medical School, Boston, Massachusetts, United States of America; 2 Department of Genetics, Harvard Medical School, Boston, Massachusetts, United States of America; 3 College of Life Science and Technology, Huazhong University of Science and Technology, Wuhan, China; 4 Research Programs Unit, Molecular Cancer Biology, and Institute of Biomedicine, Biomedicum Helsinki, University of Helsinki, Helsinki, Finland; Stanford University Medical Center, United States of America

## Abstract

SKN-1, the *Caenorhabditis elegans* Nrf1/2/3 ortholog, promotes both oxidative stress resistance and longevity. SKN-1 responds to oxidative stress by upregulating genes that detoxify and defend against free radicals and other reactive molecules, a SKN-1/Nrf function that is both well-known and conserved. Here we show that SKN-1 has a broader and more complex role in maintaining cellular stress defenses. SKN-1 sustains expression and activity of the ubiquitin-proteasome system (UPS) and coordinates specific protective responses to perturbations in protein synthesis or degradation through the UPS. If translation initiation or elongation is impaired, SKN-1 upregulates overlapping sets of cytoprotective genes and increases stress resistance. When proteasome gene expression and activity are blocked, SKN-1 activates multiple classes of proteasome subunit genes in a compensatory response. SKN-1 thereby maintains UPS activity in the intestine *in vivo* under normal conditions and promotes survival when the proteasome is inhibited. In contrast, when translation elongation is impaired, SKN-1 does not upregulate proteasome genes, and UPS activity is then reduced. This indicates that UPS activity depends upon presence of an intact translation elongation apparatus; and it supports a model, suggested by genetic and biochemical studies in yeast, that protein synthesis and degradation may be coupled processes. SKN-1 therefore has a critical tissue-specific function in increasing proteasome gene expression and UPS activity under normal conditions, as well as when the UPS system is stressed, but mounts distinct responses when protein synthesis is perturbed. The specificity of these SKN-1–mediated stress responses, along with the apparent coordination between UPS and translation elongation activity, may promote protein homeostasis under stress or disease conditions. The data suggest that SKN-1 may increase longevity, not only through its well-documented role in boosting stress resistance, but also through contributing to protein homeostasis.

## Author Summary

The mechanisms through which organisms defend against environmental stresses are critical during diverse disease processes and are likely to be important for longevity. The nematode *C. elegans* is advantageous for genetic analysis of how stress defenses function and contribute to survival. The evolutionarily conserved *C. elegans* protein SKN-1 promotes stress resistance and longevity, and it defends against toxic small molecules. We now report that in certain tissues SKN-1 also maintains production of the proteasome, a structure that degrades proteins in a regulated fashion. SKN-1 mounts distinct stress responses to perturbations in protein synthesis and degradation, in which it boosts proteasome levels only in response to proteasome impairment. Remarkably, proteasome activity also depends upon the proper functioning of the protein synthesis apparatus. The specificity of SKN-1 stress responses may be important for protein homeostasis, allowing SKN-1 to maintain levels and activity of the proteasomal degradation apparatus, but not increase degradation when protein synthesis is impaired. This role of SKN-1 in regulating protein turnover may be important for many of its stress defense functions and for protection against disease and aging.

## Introduction

Maintenance of protein homeostasis is critical for organismal health, and protection against environmental challenges. Protein homeostasis depends upon the balance among the processes of protein synthesis, folding, and degradation. Disruptions in this balance result in accumulation of abnormal proteins, which over time leads to deterioration of cellular functions, and ultimately to cell death [1], [2]. Imbalances in proteostasis are central to progression of numerous disorders, including some cancers, neurodegenerative and alcoholic liver disease, and type 2 diabetes [3], [4].

Most intracellular proteolysis is mediated by the 26S proteasome, a multicatalytic protease that degrades polyubiquitinated proteins [5]. The ubiquitin-proteasome system (UPS) regulates the stability of proteins involved in a wide range of cellular processes [6]. The 26S proteasome is composed of two subcomplexes: a barrel-shaped 20S catalytic core structure, and a 19S regulatory particle that caps it at either or both ends. The 19S regulatory particle facilitates the entry of polyubiquitinated proteins, and is composed of base and lid subcomplexes [6], [7]. It is a major challenge to understand how the levels and activity of the proteasome are regulated to maintain the balance of protein synthesis and degradation.

Several lines of evidence indicate that the proteasome associates with the mRNA translation machinery, and that the processes of protein synthesis and degradation may be linked. Proteins are synthesized through the steps of translation initiation, elongation, and termination. The elongation cycle adds amino acids to a growing polypeptide chain, and requires a set of translation elongation factors (TEFs) (Figure S1; Table S1). The elongation process is regulated through phosphorylation of TEFs in response to growth and nutrient availability signals [8]. In addition, some TEFs are involved in functions besides translation. The elongation factor eEF1A binds to proteasome subunits and ubiquitinated proteins, and thereby seems to promote degradation of damaged nascent proteins [7], [9]–[11]. Given that up to 30% of nascent polypeptides may be degraded cotranslationally [12], [13], this interaction could be important for protein quality control and homeostasis. Consistent with this idea, in *S. pombe* translation initiation factors (TIFs), TEFs, and the proteasome associate together within a “translasome” supercomplex that is proposed to facilitate degradation of defective newly-synthesized proteins [14].

Nrf1/2/3 (NF-E2-related factor) proteins defend against oxidative and xenobiotic stress by regulating transcription of numerous cytoprotective genes [15]. Recent evidence indicates that Nrf proteins also promote proteasome gene expression in some cellular contexts. Proteasome activity is increased in many cancers, and it has been shown that in colon cancer cells Nrf2 upregulates proteasome expression and activity, and thereby seems to protect against apoptosis [16]. In cultured cell lines, Nrf1 and possibly Nrf2 mobilize a compensatory “bounce-back” response in which proteasome subunit genes are upregulated when the proteasome is inhibited [17]–[19]. These findings have important implications for development of cancer therapeutics that target the proteasome, because concomitant inhibition of Nrf proteins might enhance their effectiveness [18]. Nrf1 seems to have a relatively minor role in steady state proteasome gene expression, however, raising the question of how proteasome activity might normally be fine-tuned by Nrf proteins or other mechanisms *in vivo*.

In *C. elegans*, the Nrf1/2/3 ortholog SKN-1 defends against various stresses, and upregulates expression of a wide range of cellular defense, metabolism, and repair genes under either normal or stress conditions [20], [21]. Several proteasome subunit genes are among those that appear to be regulated by SKN-1 [20], [21], and a recent genome-scale chromatin immunoprecipitation (ChIP) analysis detected SKN-1 at the promoters of most proteasome genes under non-stressed conditions during the L1 larval stage [22]. Taken together, these findings raise the possibility that SKN-1/Nrf proteins might have a conserved and essential function in regulating proteasome synthesis *in vivo*, even under normal conditions. Furthermore, in a recent screen our lab identified genes for which RNA interference (RNAi) resulted in constitutive expression of stress-inducible SKN-1 targets [23]. These genes include several involved in protein folding or degradation, the TEF *eef-1B.1*, and some TIFs. RNAi against multiple TIFs resulted in a SKN-1-dependent transcriptional response that increased stress resistance and lifespan [23]. Together, these results suggest that SKN-1 might defend against perturbations in either protein synthesis or degradation.

In this study, we have investigated how impairment of translation elongation influences the activity of SKN-1 and the proteasome, and how SKN-1 and the translation machinery affect the proteasome. We show that distinct but overlapping sets of SKN-1 target genes are induced when translation initiation or elongation is inhibited. In the intestine, which is the *C. elegans* counterpart to the gut, liver, and adipose tissue, SKN-1 mediates a bounce-back response to proteasome gene inhibition, and also maintains UPS activity *in vivo* under normal conditions. Importantly, impairment of translation elongation does not induce this bounce-back response, and instead reduces intestinal UPS activity. The data reveal a remarkable degree of complexity in how SKN-1/Nrf proteins respond to different stresses, and suggest that the functional relationships between the translation elongation apparatus, SKN-1/Nrf proteins, and the proteasome are important for protein homeostasis.

## Results

### Induction of distinct SKN-1–mediated stress responses by inhibition of translation initiation or elongation

**Figure 1:**
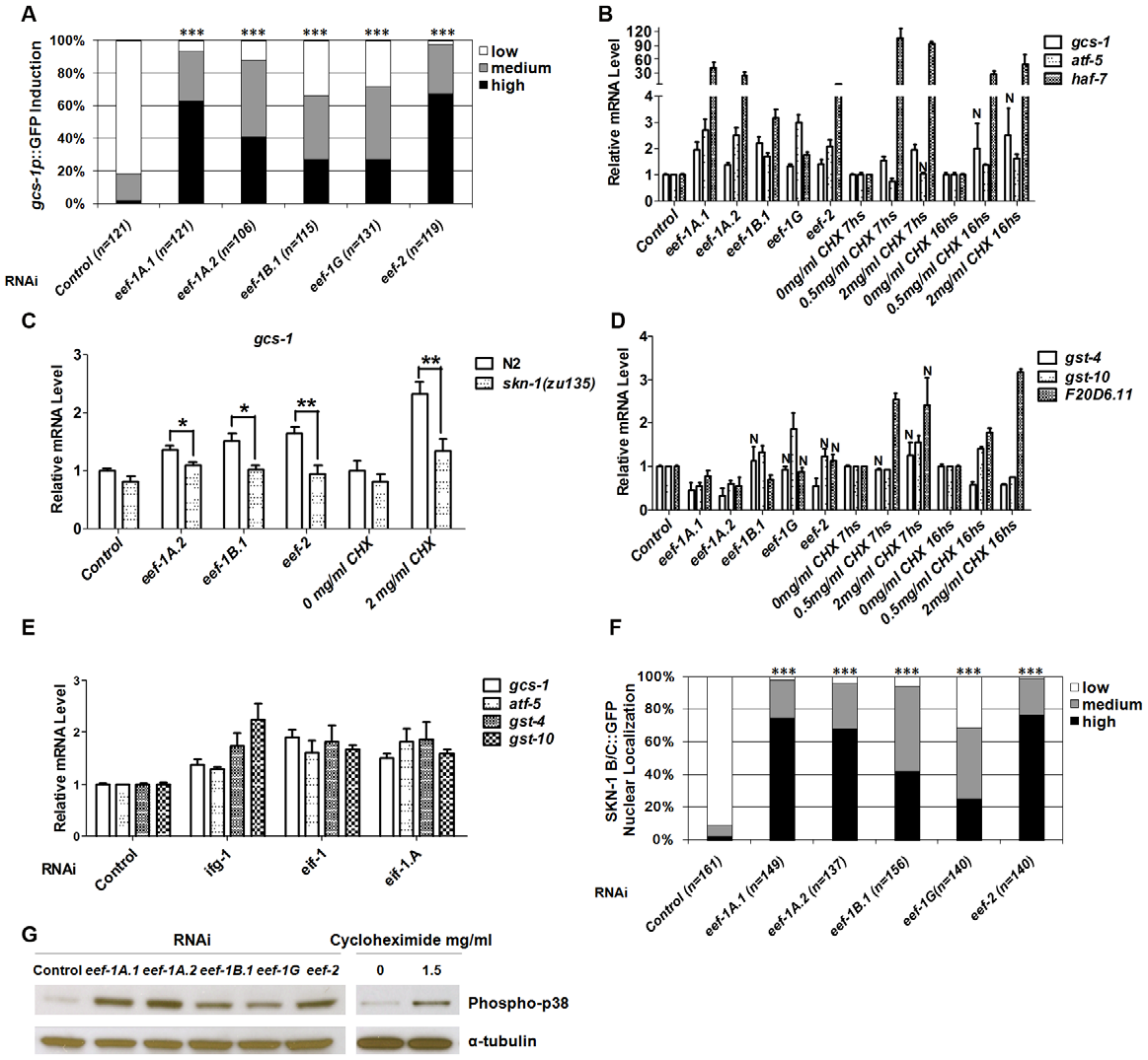
Induction of a SKN-1–mediated stress response by interference with translation elongation. (A) The SKN-1-regulated reporter *gcs-1*p::GFP is activated by TEF RNAi. GFP levels were scored in the anterior intestine by a system similar to that we have described previously [24], with unambiguously bright levels defined as “High”, barely-detectable GFP levels scored as “Low”, and medium expression being intermediate between “Low” and “High”. Here and in (F), *P* values were derived from a chi^2^ test, *** *P*<0.0001. (B) Induction of endogenous SKN-1 target genes by interference with translation elongation. mRNA levels relative to control are shown, detected by quantitative Real Time PCR (qRT-PCR) after TEF RNAi or CHX treatment (for 7 or 16 hours (hs)). Here and in (E), *P*<0.05 compared to control except where not significant is indicated by N. Error bars indicate SEM. (C) SKN-1-dependence of target gene induction. Endogenous *gcs-1* mRNA was detected by qRT-PCR in wild-type (N2) or *skn-1(zu135)* animals that had been fed with TEF RNAi bacteria, or treated with CHX for 18 hr at 15°C. Differences between wild-type (N2) and *skn-1(zu135)* animals were analyzed by paired t test (two-tailed). In all qRT-PCR experiments where asterisks are shown, ***P<0.001, **P<0.01, *P<0.05. An unpaired t test (two-tailed) was used to compare effects of TEF RNAi or CHX treatment with the corresponding N2 control. For each comparison to N2, *P*<0.001. (D) Interference with translation elongation does not upregulate all SKN-1 target genes. Endogenous SKN-1 target mRNA levels were assayed by qRT-PCR after TEF RNAi or CHX treatment. (E) Induction of SKN-1 target genes by TIF RNAi, assayed by qRT-PCR. (F) SKN-1 accumulates in intestinal nuclei in response to TEF RNAi. Examples of nuclear SKN-1 B/C::GFP scoring are shown in Figure S2E. (G) Activation of p38 MAPK signaling in response to inhibition of translation elongation. Lysates from worms that were exposed to TEF RNAi or CHX (13 hs) were analyzed by Western blotting for phosphorylated (active) p38 kinase.

To investigate whether SKN-1 activity is generally influenced by translation elongation, we performed RNAi against 5 of the 7 predicted *C. elegans* TEFs (Table S1). We first monitored expression of a transgene in which the promoter for the SKN-1 target gene *gcs-1* (γ-glutamyl cysteine synthetase) is fused to green fluorescent protein (GFP) (Figure S2A) [24]. RNAi against each TEF upregulated *gcs-1p*::GFP in the anterior intestine (Figure 1A) and increased expression of endogenous *gcs-1* mRNA (Figure 1B). Mutation of an important SKN-1 binding site (Figure S2A) [24] diminished *gcs-1* promoter induction (Figure S2B), and upregulation of endogenous *gcs-1* mRNA was eliminated in a *skn-1* mutant (Figure 1C), indicating that SKN-1 was required for *gcs-1* induction in response to TEF RNAi.

We next investigated how TEF knockdown influences expression of other SKN-1 target genes. The SKN-1-dependent genes *atf-5* and *haf-7* [20], [23] were upregulated in a manner that was either partially or completely dependent upon *skn-1* (Figure 1B and Figure S2C). In contrast, the SKN-1 targets *gst-4*, *gst-10* and *F20D6.11* were generally not induced in response to TEF RNAi (Figure 1D and Figure S2D). This was surprising, because *gst-4* is upregulated by SKN-1 under normal conditions, in response to various stresses, and after inhibition of insulin-like signaling (IIS) or translation initiation [20], [23], [25]–[27]. Similarly, *gst-10* and *F20D6.11* are induced by SKN-1 in response to reduced IIS and TIF RNAi, respectively [23], [25]. We further compared effects of translation initiation and elongation by analyzing animals subjected to RNAi against the TIFs *ifg-1* (eIF4G), *eif-1* (eIF-1) and *eif-1.A* (eIF-1A). In contrast to the effects of TEF RNAi, RNAi against these TIFs consistently upregulated endogenous *gst-4* and *gst-10*, along with *gcs-1* and *atf-5* (Figure 1E and Figure S2D). Taken together, our data indicate that SKN-1 upregulates overlapping but distinct sets of target genes in response to inhibition of translation initiation or elongation.

Cycloheximide (CHX) blocks translation elongation by competing with the binding of ATP to the 60S ribosomal subunit, and inhibiting eEF2-mediated translocation (Figure S1) [28]. Treatment with CHX generally mimicked the effects of TEF RNAi on SKN-1 target gene expression, except that *F20D6.11* was also upregulated (Figure 1B and 1D). This suggests that a SKN-1-dependent stress response is induced by inhibition of the translation elongation process *per se*, not simply by a lack of TEFs.

We next investigated how TEF knockdown influences the levels of SKN-1 in intestinal nuclei. A transgenic protein that includes two SKN-1 isoforms fused to GFP (SKN-1 B/C::GFP) readily accumulates in intestinal nuclei in response to various stresses, or reductions in IIS [24]–[26]. TEF knockdown also dramatically increased SKN-1 accumulation in intestinal nuclei, without upregulating endogenous *skn-1* transcripts, indicating that elongation inhibition increases SKN-1 nuclear accumulation post-transcriptionally (Figure 1F, Figure S2E and S2F). In striking contrast, TIF RNAi does not detectably increase the overall levels of nuclear SKN-1 [23]. TIF inhibition therefore appears to upregulate SKN-1 target genes through a different mechanism, and may act on processes that cooperate with SKN-1 but do not influence its nuclear accumulation.

The evolutionarily conserved p38 mitogen-activated protein kinase (MAPK) signaling pathway is required for oxidative stress to induce SKN-1 nuclear accumulation and target gene activation [29]. The activity of this pathway can be assessed in *C. elegans* by Western blotting for the dually phosphorylated, active form of p38 kinase [29], [30]. We observed that both TEF RNAi and CHX treatment dramatically elevated the levels of phospho-p38 (Figure 1G). This signal and *gcs-1* promoter induction were markedly reduced in the MAPKK and MAPKKK null mutants *sek-1(km4)* and *nsy-1(ok593)* respectively, indicating that the canonical p38 pathway was required (Figure S2G and S2B, respectively). With the exception of *ifg-1*, RNAi against TIFs did not robustly activate *sek-1*-dependent p38 MAPK activity, further supporting the idea that TIFs and TEFs influence SKN-1 activity largely through distinct mechanisms (Figure S2H).

### Impaired translation elongation does not induce stress defenses non-specifically

**Figure 2:**
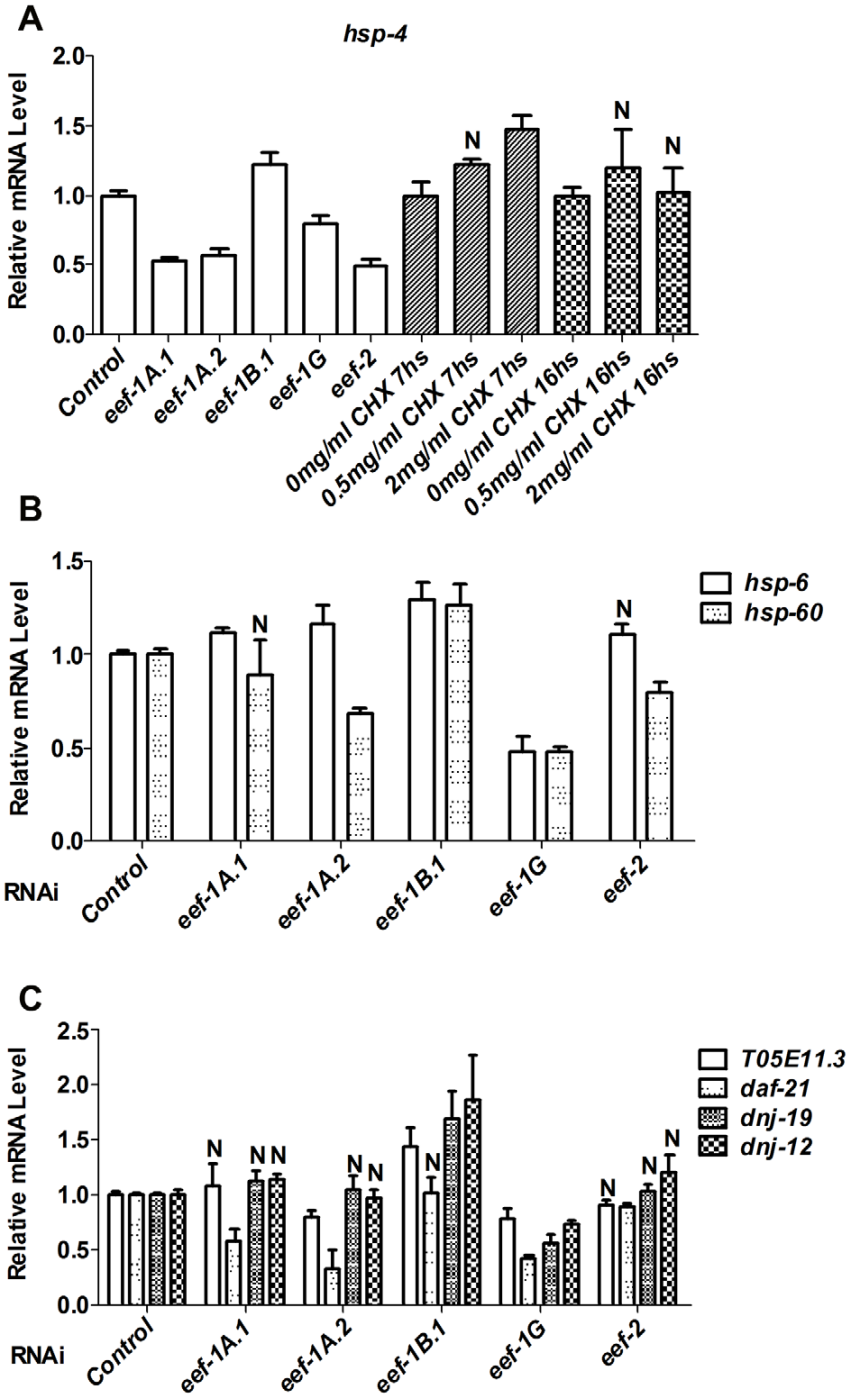
TEF inhibition does not globally induce stress responses. In (A–C), Relative levels of the indicated endogenous mRNAs were assayed by qRT-PCR after RNAi against the indicated TEF, or CHX treatment. N = not significant. In (A), all other *P*<0.025; in (B), all other *P*<0.05; in (C), all other *P*<0.02.

It is an important question whether the SKN-1-mediated response to reduced translation elongation might derive simply from a non-specific activation of multiple stress defenses. To test this idea, we investigated how other stress responses involved in protein homeostasis are influenced by TEF RNAi. An accumulation of misfolded proteins in the endoplasmic reticulum (ER) or mitochondria triggers the ER unfolded protein response (UPR^er^), or mitochondrial UPR (UPR^mt^), respectively. To investigate whether the UPR^er^ or UPR^mt^ is activated in response to inhibition of TEFs, we examined transcriptional levels of *hsp-4*, an indicator of the UPR^er^ [31], along with the UPR^mt^ indicators *hsp-6* and *hsp-60* [32]. In general, TEF RNAi did not robustly increase the levels of *hsp-4*, *hsp-6*, or *hsp-60* mRNAs (Figure 2A and 2B). Heat shock proteins (HSPs) act as chaperones that cope with misfolded cytoplasmic proteins during multiple stresses. As an indicator of effects on this network, we assayed for induction of genes representing four major classes of heat shock proteins: small HSPs (*hsp-16.2*), DnaJ/HSP40s (*dnj-19*, *dnj-12*), Hsc/HSP70s (*hsp-70*), and HSP90s (*T05E11.3*, *daf-21*) [33]. These HSP genes were also not upregulated in response to inhibition of most TEFs (Figure 2C, Figure S3A and S3B). Taken together, the data indicate that RNAi against TEFs does not broadly activate stress responses involved in proteostasis.

In *C. elegans*, interference with translation initiation or elongation decreases brood size (Figure S3C) [34]–[36], raising the concern of whether the activation of SKN-1 that results from TEF RNAi might derive in part from reduction in germline proliferation. Interference with germ cell proliferation stimulates translocation of the transcription factor DAF-16/FOXO into intestinal nuclei, resulting in increased DAF-16 target gene expression and a *daf-16*-dependent increase in longevity [37], [38]. In contrast, TEF RNAi only minimally affected either DAF-16 nuclear levels, or expression of the DAF-16 target *sod-3* (Figure S3D and S3E). Interference with germ cell proliferation also dramatically upregulated expression of the SKN-1 target *gst-4* (Blackwell lab, unpublished), which is not induced by TEF RNAi (Figure 1D and Figure S2D). Together, these results suggest that the effects of translation elongation inhibition on SKN-1 activity do not derive from either a non-specific stress response, or indirect effects mediated by the germline.

### SKN-1 increases stress resistance in response to reduced translation elongation

**Figure 3:**
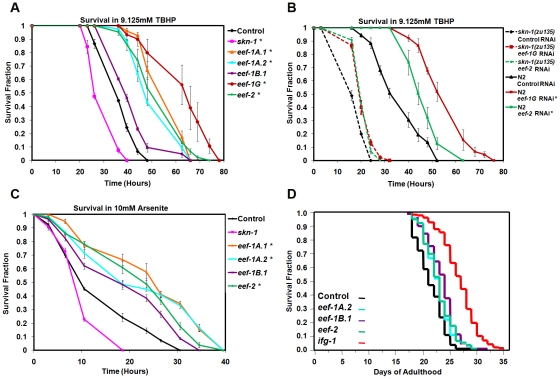
TEF knockdown increases oxidative stress resistance. (A) Increased TBHP resistance after TEF RNAi. In (A–C), data were analyzed by JMP and plotted with EXCEL. Representative experiments are shown, with replicates, statistics, and percent changes in survival time provided in Table S2 (A), Table S3 (B), and Table S4 (C). Error bars represent the SEM, and *P* values were calculated by log-rank. *P<0.0001. (B) *skn-1* contributes to TBHP resistance deriving from TEF RNAi. (C) Resistance to Arsenite resulting from TEF RNAi. (D) Lifespan analysis of worms fed TEFs or TIF (*ifg-1*) RNAi bacteria. A composite of three replicates (Table S5A) is shown.

RNAi against TIF or ribosomal protein genes increases resistance to various environmental stresses [23], [34]–[36]. We therefore examined whether TEF knockdown affects resistance to two different sources of oxidative stress, the organic hydroperoxide tert-butyl hydrogen peroxide (TBHP), and the metalloid sodium arsenite (As) [20]. TBHP resistance was dramatically increased after knockdown of multiple TEFs in wild type animals (Figure 3A; Table S2). In contrast, RNAi against *eef-2* or *eef-1G* did not robustly increase oxidative stress resistance in *skn-1(zu135)* mutants, indicating that *skn-1* is essential for the TBHP resistance that derives from TEF knockdown (Figure 3B; Table S3). TEF inhibition also increased resistance to As (Figure 3C; Table S4). We conclude that the SKN-1-mediated transcriptional response to impaired translation elongation increases oxidative stress resistance.

*C. elegans* lifespan is increased by mutation or adulthood knockdown of several TIFs, ribosomal proteins, or other translation regulators [23], [34], [35]. TIF and TEF mRNAs are expressed at lower levels in the long-lived IIS mutant *daf-2*, also consistent with an opposing correlation between protein synthesis and longevity [39]. However, when we performed TEF RNAi by feeding during adulthood, lifespan was increased slightly by knockdown of *eef-1A.2*, *eef-1B.1* and *eef-2*, but not by *eef-1A.1* or *eef-1G* (Figure 3D; Table S5A; Figure S3F; Table S5B). This failure of TEF RNAi to increase lifespan robustly could arise from TEF RNAi having more pleiotropic effects on the animal than TIF knockdown, or could be related to the differences in gene expression responses that result from interference with translation elongation and initiation.

### SKN-1 mediates a proteasome bounce-back response and maintains UPS activity tissue-specifically *in vivo*

For multiple reasons, we examined the involvement of SKN-1 in proteasome gene regulation and activity. Firstly, our microarray-based expression profiling suggested that SKN-1 contributes to transcription of 14 proteasome subunit genes (44% of the apparent total), under both normal and oxidative stress conditions [20]. Secondly, a transgenic SKN-1::GFP fusion protein was detected with high confidence at the promoter regions of 25 proteasome genes (78% of the apparent total) during the L1 larval stage [22]. These included all of the proteasome genes that expression profiling suggested are regulated by SKN-1, with only a single exception (*rpt-5*). In addition, some SKN-1 target genes are induced by RNAi knockdown of proteasome genes [23], [26], [27]. Finally, as suppression of translation elongation might increase the fraction of incompletely translated proteins, it seemed possible that SKN-1 might increase proteasome gene expression and activity in response to interference with translation elongation.

**Figure 4:**
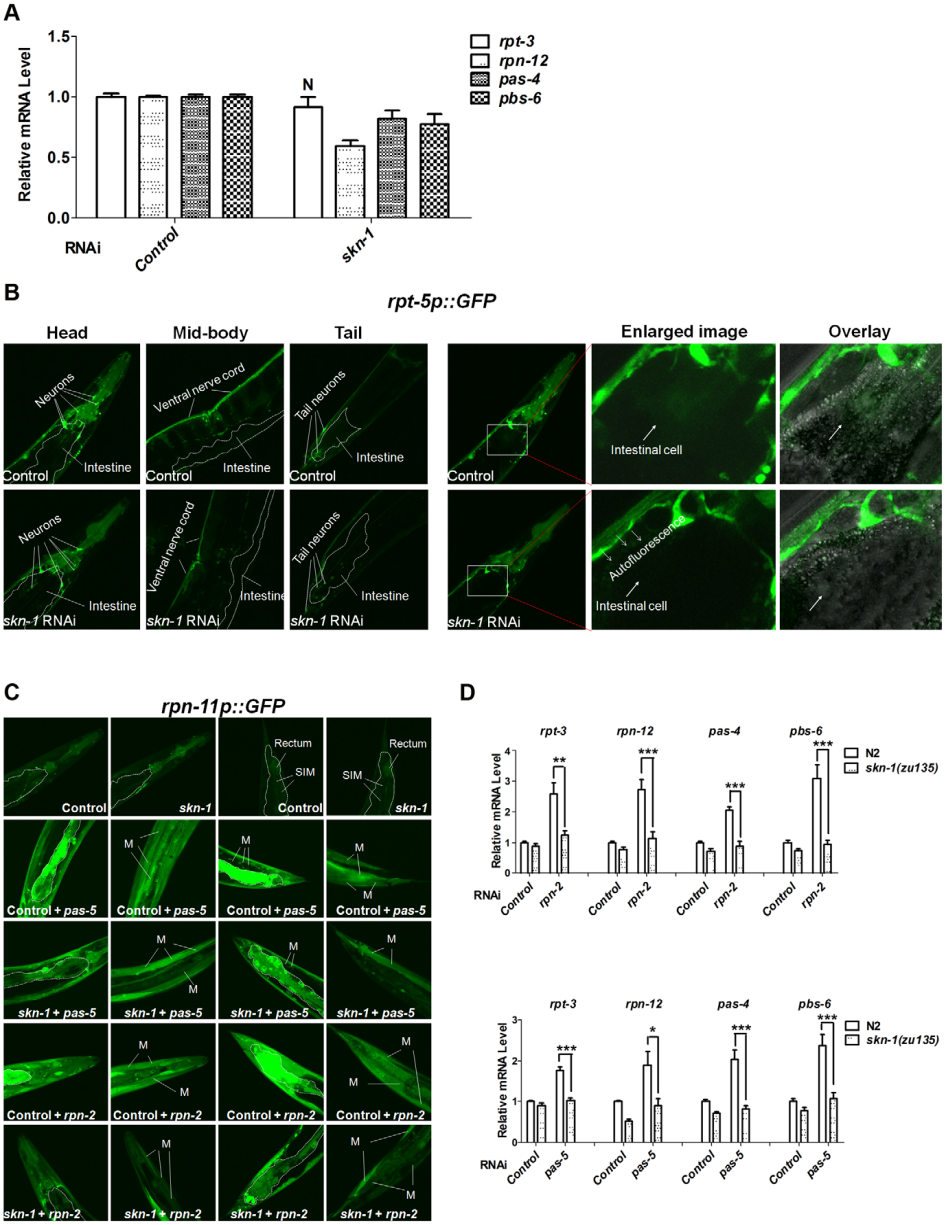
Importance of SKN-1 for the proteasome bounce-back response. (A) Relative mRNA levels of endogenous proteasome subunit mRNAs in N2 animals fed with control (L4440) or *skn-1* RNAi bacteria. All *P*<0.005 compared to control, except where N = not significant. Error bars indicate SEM. (B) *skn-1* RNAi slightly decreases *rpt-5p*::GFP expression, particularly in the intestine, under normal conditions. Representative projection images show all z-stacks in the left panels, and z-stacks through the intestine on the right. Here and in (C), 2-day-old adults were used for imaging, boundaries of the intestine are indicated by dashed lines, and quantification and statistics are listed in Table S6. (C) *skn-1* RNAi blocks the bounce-back response to proteasomal subunit knockdown. Representative confocal projection images of *pas-5* or *rpn-2* RNAi effects on the *rpn-11p::GFP* transcriptional reporter are shown, with z-stacks through the intestine or muscles displayed for double RNAi experiments. Abbreviations: M, body-wall muscle; SIM, stomatointestinal muscle. (D) Impaired bounce-back response in *skn-1* mutants. Endogenous proteasome subunit mRNAs were detected by qRT-PCR in wild-type (N2) or *skn-1(zu135)* animals that had been fed with proteasome subunit RNAi bacteria. A paired t test (two-tailed) was employed to compare wild-type (N2) and *skn-1(zu135)* animals. An unpaired t test (two-tailed) was used to compare proteasome subunit vs control RNAi in N2 animals. Compared to N2 control, all *P*<0.05. mRNA levels were normalized to *tba-1* (α-tubulin).

We first investigated the extent to which *skn-1* is required for proteasome gene expression under normal conditions. The 26S proteasome consists of at least 32 subunits in *C. elegans*, including 19S ATPases involved in substrate unfolding (*rpt-1∼6*), other 19S subunits (*rpn-1∼12*), 20S α-rings (*pas-1∼7*) and 20S β-rings (*pbs-1∼7*) [11]. We examined how *skn-1* RNAi affected the expression of the endogenous proteasome subunit genes *rpt-3*, *rpn-12*, *pas-4* and *pbs-6*, which represent the four subunit classes above. Each of these genes is a predicted SKN-1 target at which at least four canonical SKN-1 binding sites lie within 1 kb upstream of the translation initiation codon, and SKN-1::GFP was detected by ChIP [20], [22] (data not shown). In whole animals *skn-1* RNAi slightly decreased the expression of each gene, except for *rpt-3* (Figure 4A). We also examined expression of transcriptional reporters in which proteasome promoters are fused to GFP. RNAi against *skn-1* slightly decreased expression of reporters for *rpt-5*, *rpn-11 and pas-5*, particularly in the intestine, but did not detectably affect *rpn-2* or *pbs-4* (Figure 4B and 4C, Figure S4A and S4B, Table S6). The data suggest that under normal conditions SKN-1 contributes to but is apparently not essential for the expression of many proteasome subunit genes.

In mammalian cells, Nrf1 and Nrf2 have been implicated in the “bounce-back” response whereby inhibition of the proteasome results in a compensatory upregulation of proteasome subunit gene expression [17]–[19]. To test this model in *C. elegans* tissues *in vivo*, we blocked proteasome activity by performing RNAi against an essential proteasome subunit gene, then examined expression of other proteasome genes. Knockdown of *pas-5* or *rpn-2* resulted in dramatic upregulation of the *pbs-4*, *rpt-5* and *rpn-11* transcriptional reporters, as well as an RPN-11::GFP translational fusion protein (Figure 4C, Figure S4A and S4B, Table S6). These increases in proteasome gene expression were largely dependent upon *skn-1* in the intestine, where SKN-1 is prominently expressed [24], as well as in some muscles. Additionally, *pas-5* or *rpn-2* knockdown increased endogenous proteasome subunit mRNA levels in a *skn-1*-dependent manner (Figure 4D). In certain tissues, therefore, SKN-1 is required *in vivo* for the compensatory induction of proteasome gene upregulation that occurs in response to proteasome inhibition.

**Figure 5:**
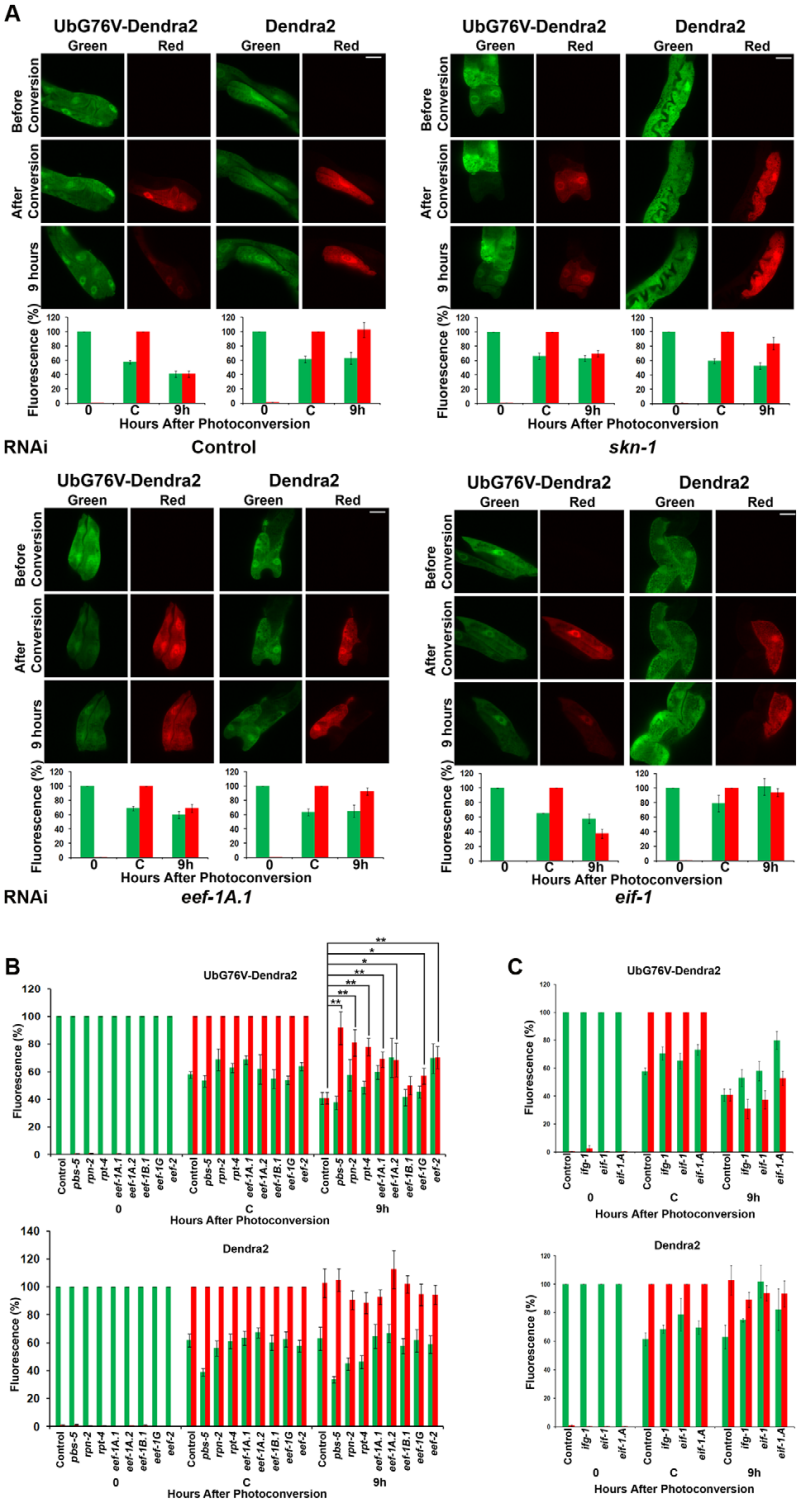
Knockdown of SKN-1 or TEFs, but not TIFs, reduces UPS activity in the intestine. (A) Representative images of *Pvha-6::UbG76V-Dendra2 and Pvha-6::Dendra2* reporters in animals exposed to control, *skn-1*, *eef-1A.1* (TEF) or *eif-1*(TIF) RNAi. Red fluorescence derives from substrate that was present at the time of photoconversion. Bar: 20 µm. In (A–C), bar graphs depict the percentages of green and red fluorescence compared to either the initial value (t = 0 for green) or point of photoconversion (t = C for red) for UbG76V-Dendra2 and Dendra2, respectively (± SEM). (B,C) Summary of UbG76V-Dendra2 and Dendra2 imaging in intestinal cells after 9 hours in response to TEF and TIF RNAi, respectively. Significant differences relative to control RNAi (red fluorescence after 9 hours) are indicated with **P<0.01, *P<0.05.

As our data suggested that *skn-1* contributes to proteasome gene expression, particularly when proteasome activity is impaired, we used a novel *in vivo* assay to investigate whether SKN-1 is important for UPS activity under normal conditions [40]. We generated a strain (*Pvha-6::UbG76V-Dendra2*) in which the intestine-specific promoter *vha-6* drives expression of a photoswitchable green-to-red fluorescent protein (Dendra2) that is fused to a non-hydrolyzable ubiquitin moiety (UbG76V) [40], [41]. By monitoring this fusion protein after photoconversion, we could assess ubiquitin-dependent protein degradation activity in living animals [40]. In control RNAi animals, at 9 hours after photoconversion the levels of red-fluorescing intestinal UbG76V-Dendra2 had been reduced to 40% of that present just after photoconversion, but a control Dendra2 that lacked UbG76V was still stable (Figure 5A, upper left panels). UbG76V-Dendra2 degradation was dramatically inhibited by RNAi against the proteasome genes *pbs-5*, *rpn-2*, or *rpt-4*, indicating that this degradation required the proteasome (Figure 5B and S4C). Together, the data show that this intestinal UbG76V-Dendra2 protein is degraded by the UPS.

Degradation of intestinally-expressed UbG76V-Dendra2 was also markedly reduced by *skn-1* RNAi, indicating that UPS activity in the intestine depends upon SKN-1 (Figure 5A, upper panels). In contrast, *skn-1* RNAi did not impair degradation of UbG76V-Dendra2 that was expressed specifically in the body-wall muscle, and slightly increased its degradation in dopaminergic neurons (Figure S4D and S4E, respectively). *skn-1* RNAi also decreased the total proteasome activity in the animal under normal conditions, as detected *in vitro* by a proteasome in-gel activity assay (Figure S5A). Treatment with the proteasome inhibitor MG132 was more toxic for animals in which *skn-1* had been knocked by RNAi than for control animals (Figure S5B), further supporting the idea that SKN-1 is important for proteasome gene expression and activity. We conclude that SKN-1 functions tissue-specifically to maintain UPS activity in the intestine under normal conditions, and that a significant proportion of total *C. elegans* UPS activity is *skn-1*-dependent.

### Dependence of the UPS on translation elongation factors

Having determined that SKN-1 is important for proteasome gene expression and UPS activity in the intestine, and that RNAi against TEFs induces SKN-1 to upregulate particular target genes, we wanted to investigate whether interference with translation elongation might direct SKN-1 to increase proteasome expression and degradation activity. This seemed like a plausible model, because it might be advantageous for proteasome activity to be increased upon interference with elongation, in order to ensure that any incompletely translated proteins are degraded. Surprisingly, however, the levels of endogenous mRNAs encoding four proteasome subunits decreased slightly in response to RNAi against each TEF that we examined, with the exception of *eef-1B.1* (Figure S6A). Intestinal fluorescence from the *pas-5p::GFP* reporter was not increased by either *eef-1A.1* or *eef-2* RNAi, but was slightly decreased by *eef-2* knockdown (Figure S6B; Table S6). Furthermore, in whole animals the levels of proteasome 20S α subunits were decreased slightly in response to RNAi against each TEF (Figure S6C). Expression of proteasome subunits was similarly reduced slightly by RNAi against TIFs (Figure S6A and S6D). We conclude that whereas SKN-1 directly upregulates proteasome gene transcription under normal conditions, and particularly after depletion of individual proteasome subunits, it does not do so after inhibition of translation elongation or initiation.

In yeast, eEF1A interacts with proteasome subunits and may escort incompletely translated proteins to the proteasome, thereby facilitating their degradation [7], [9], [10]. This raised an alternative possibility, that the translation elongation apparatus might be important for proteasome activity. Consistent with this notion, in *C. elegans* EEF-1A.1 interacts with proteasome subunits RPN-2 and RPT-4 [11] and inhibition of three TEFs resulted in the premature aggregation of transgenic proteins, suggesting a possible downregulation of proteasome activity [42]. When we monitored UbG76V-Dendra2 degradation in the intestine, we observed that its degradation was significantly impaired by knockdown of multiple different TEFs, but not TIFs (Figure 5; Table S7). Having observed that proteasome gene expression is affected similarly by TEF and TIF RNAi (Figure S6A–S6D), this suggests that UPS activity may be mechanistically dependent upon the translation elongation machinery.

These findings raised an unexpected model for why SKN-1 target genes are induced by RNAi against TEFs: that the resulting reduction in proteasome activity might stimulate a SKN-1-dependent stress response. However, several observations argue against this interpretation. In contrast to the effects of TEF RNAi, knockdown of proteasome subunit genes induced *skn-1*-dependent expression of other proteasome genes, and did not increase p38 MAPK signaling or SKN-1 nuclear occupancy (Figure 4C, 4D and S6E–S6G; Table S6). Also different from TEF RNAi effects, proteasome gene RNAi activated the *skn-1*-regulated *gst-4p::GFP* reporter [26], [27], and knockdown of *pas-5*, *rpn-2* or *rpt-4* induced *skn-1*-dependent endogenous *gst-4* and *gst-10* expression (Figure S6G and S6H). Finally, proteasome subunit but not TEF RNAi activated heat shock promoters *hsp-70* and *hsp-16.2* (Figure S3A and S3B). Induction of a SKN-1-mediated stress response by TEF RNAi therefore does not derive from an indirect effect on the proteasome, and may result directly from signals associated with slowed translation elongation.

## Discussion

**Figure 6:**
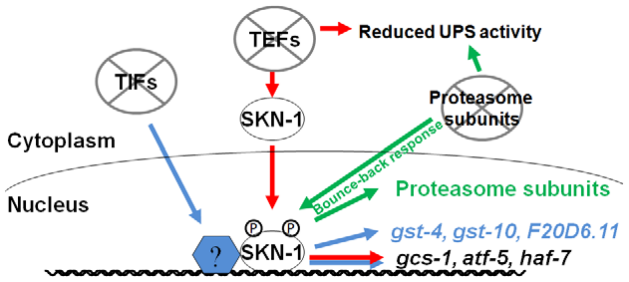
Specific SKN-1–mediated responses to impaired protein synthesis and degradation. SKN-1 target genes are activated through distinct mechanisms by interference with translation initiation or elongation, so that TEF RNAi (red) induces a more restricted set of SKN-1 target genes than TIF RNAi (blue). TEF RNAi also impairs UPS activity, supporting the idea that proteasome activity and translation elongation are mechanistically coupled processes. In the intestine, SKN-1 is also important for the “bounce-back” response to proteasome inhibition, and for UPS activity under normal conditions (green).

We have determined that interference with either mRNA translation or proteasome integrity results in induction of SKN-1-mediated stress responses. These responses are remarkably specific, in that SKN-1 upregulates distinct suites of target genes in response to impairment of translation initiation, translation elongation, or proteasome activity (Figure 6). When protein synthesis is inhibited, SKN-1 increases oxidative stress resistance. If proteasome subunit expression is blocked, SKN-1 attempts to compensate by upregulating proteasome genes in multiple tissues. In contrast, proteasome gene expression is not increased when translation is impaired, and proteasome activity is actually decreased in response to reduced translation elongation, suggesting that the specificity of these SKN-1-mediated functions may be important for maintaining protein homeostasis.

It is intriguing that different mechanisms seem be involved when SKN-1 is directed to activate target genes in response to inhibition of translation initiation or elongation. It is unlikely that the differences between these SKN-1-dependent responses derive simply from different degrees of translation activity or stress, because these responses are qualitatively distinct. Whereas TIF but not TEF RNAi upregulates transcription of the SKN-1 target genes *gst-4* and *gst-10* (Figure 1B, 1D and 1E), TEF but not TIF RNAi leads to accumulation of SKN-1 in intestinal nuclei (Figure 1F) [23]. In addition, TEF RNAi increases p38 pathway signaling more robustly (Figure 1G and Figure S2G). One possible model is that interference with translation initiation might upregulate SKN-1-dependent gene expression by acting on transcription factors that cooperate with SKN-1. Consistent with this idea, several mRNAs are translated preferentially when translation initiation is inhibited, including some that encode stress response factors [43]. It may be important to increase oxidative and xenobiotic stress resistance when either translation initiation or elongation is impaired, because broad reductions in protein synthesis could disrupt cellular metabolism or redox buffering, particularly in a key metabolic and synthetic tissue like the *C. elegans* intestine [23]. In addition, oxidizing conditions facilitate IIS, suggesting that under conditions of growth and high translation rates it could be advantageous to suppress SKN-1-regulated oxidative stress defenses [44].

In addition to its well-documented role in small molecule detoxification, we have found that SKN-1 is also important for regulating proteasome gene expression and sustaining UPS activity, particularly in the intestine. The SKN-1 orthologs Nrf1 or Nrf2 have been reported to induce compensatory proteasome gene expression when proteasome activity is impaired in cultured mammalian cells [17]–[19]. We have shown that this SKN-1/Nrf function is both important *in vivo* and evolutionarily conserved, and involves each class of proteasome genes. We also obtained the novel finding that SKN-1 orchestrates this response in multiple post-mitotic tissues, including the intestine. It will be important to investigate the extent to which the proteasome bounce-back response might rely on different Nrf1/2/3 isoforms or other mechanisms in various mammalian cell types, particularly the gut, liver, and adipose tissues, which are counterparts to the *C. elegans* intestine.

Under non-stressed conditions, lack of SKN-1 or Nrf1 decreased proteasome gene expression only modestly in *C. elegans* and mammalian cells, respectively (Figure 4A and 4B) [18], [20]. However, this seemingly small effect of SKN-1 evidently has substantial consequences, because we determined that under normal conditions SKN-1 has a major effect on UPS activity *in vivo* in the intestine (Figure 5A, upper panels), and contributes to the total proteasome activity in the animal (Figure S5A). Taken together with recent ChIP data indicating that SKN-1 occupies the promoters of most proteasome genes under non-stressed conditions [22], our findings suggest that SKN-1/Nrf proteins are critical regulators of proteasome genes even under normal circumstances. Perhaps the “bounce-back” function of SKN-1 is needed for fine-tuning the levels of proteasomal subunits in the intestine, so that proteasome assembly can proceed efficiently.

Our observation that animals fed *skn-1* RNAi bacteria are sensitized to treatment with a proteasome inhibitor (Figure S5B) suggests that SKN-1 is critical for sustaining proteasomal defenses against proteotoxicity *in vivo*. The involvement of SKN-1/Nrf proteins in regulating proteasome gene expression might be important not only under acute stress conditions, but also in situations of chronic proteotoxic stress such as alcoholic liver and neurodegenerative diseases. Interestingly, in mice liver-specific inactivation of Nrf1 results in non-alcoholic steatohepatitis and hepatic cancer [45]. This syndrome is associated with oxidative damage in hepatocytes, but our results suggest that impaired proteasome activity might also be involved. SKN-1/Nrf proteins have been shown to increase longevity in both *C. elegans* and *Drosophila* [15], [25]. Our new results predict that this effect may derive not only from their function in protecting against reactive small molecules, but also may involve their role in sustaining proteasome expression and activity, and thereby helping to maintain protein homeostasis. Consistent with this idea, a recent study showed that *skn-1* is required for *C. elegans* lifespan to be extended by an amyloid-binding compound that suppresses toxicity deriving from misfolded proteins [46].

We also observed that UPS activity is dependent upon the translation elongation machinery. A conclusive assessment of how TEF RNAi affected total proteasome activity in the animal, as measured *in vitro*, was problematic because translation inhibition reduced the total amount of protein present (data not shown). However, our *in vivo* assay [40] demonstrated clearly that intestinal UPS activity was reduced by TEF but not TIF RNAi (Figure 5). Previous work in yeast had noted that TEFs interact with the proteasome, and that eEF1A may facilitate degradation of defective newly synthesized proteins by escorting them to the proteasome (see Introduction). Working in a metazoan, we have now obtained support for the idea that UPS-mediated protein degradation and translation elongation are mechanistically coupled processes. We determined that inhibition of translation elongation but not initiation reduced UPS activity in the intestine *in vivo*, an effect that seems unlikely to be mediated by the modest decline in proteasome gene expression seen after RNAi of either TEFs or TIFs (Figure 5 and Figure S6A–S6D). It also seems unlikely that this effect derived simply from the UPS being swamped by incompletely translated proteins arising from inhibition of elongation, because we did not see simultaneous upregulation of proteasome genes. Interestingly, our assay measured degradation of fluorescent and presumably folded UbG76V-Dendra2, suggesting that the translation elongation apparatus may promote UPS-mediated degradation of complete polypeptides that are no longer associated with the translation apparatus. The physical interactions that have been described between the proteasome and elongation factors [6], [14] therefore may be generally important for UPS activity.

It is an intriguing question why SKN-1 does not increase proteasome gene expression and activity when translation elongation is inhibited, particularly when it appears to be present at most proteasome gene promoters constitutively [22]. Perhaps it would be deleterious for SKN-1 to do so, because if translation elongation were to slow in response to limited nutrients or other conditions, an inappropriate increase in proteasome activity might prematurely degrade nascent polypeptides, and thereby could globally impair protein synthesis. This could provide a rationale not only for the failure of TEF RNAi to induce proteasome gene upregulation, but also for the apparent dependence of UPS activity on translation elongation but not initiation factors. Taken together, our findings indicate that SKN-1 plays an important role in sensing and maintaining protein homeostasis, by mobilizing distinct responses to perturbations in polypeptide chain elongation and proteasomal degradation (Figure 6). They also indicate that the stress defenses that are regulated by SKN-1/Nrf proteins are not controlled in unison through a simple on/off switch, but are remarkably customized for specific conditions. This raises important questions concerning how these stresses are sensed at the molecular level, and how different stress signals are integrated by SKN-1/Nrf proteins to achieve specificity in their responses.

## Materials and Methods

### RNAi

RNAi was performed by feeding essentially as described [23], except that L3 and (or) early L4-stage worms were fed RNAi bacteria for 3 days at 20°C unless otherwise indicated. Bacteria carrying the vector plasmid L4440 were used as the control. RNAi constructs were taken from the Vidal ORFeome-Based RNAi library [47] and confirmed by sequencing. In all double RNAi experiments, RNAi and/or control bacteria were mixed at a 1∶1 volume ratio. The wild-type strain is N2.

### RNA isolation and quantitative RT-PCR

Animals subjected to RNAi were collected and washed 3 times in M9, then total RNA was extracted from approximately 60 animals for each treatment. RNA was extracted using the TRI Reagent (Sigma) and cDNA synthesized using the SuperScript First-Strand Synthesis Kit (Invitrogen). SYBR Green Real Time Quantitative PCR was carried out using the ABI 7900 and analyzed using the Standard Curve method [48]. For all RNAi experiments, qRT-PCR data were derived from 3–4 independent biological replicates. In CHX experiments, the values presented were derived from 2–3 independent PCR analyses of one biological experiment. Results were graphed so that the level of each mRNA that was seen in N2 animals fed with control (L4440) RNAi bacteria was set as 1. Unless otherwise indicated, *act-1* (β-actin) was used for normalization and *P* values were derived from an unpaired t test (two-tailed). Primer sequences are listed in Table S8.

### Western blotting

L2/L3 stage larvae were fed RNAi bacteria for two days, then collected and washed in M9 buffer, and snap frozen in liquid nitrogen. Worms were lysed in RIPA buffer (50 mM Tris [pH = 8.0], 150 mM NaCl, 1% Nonidet P-40, 0.5% sodium deoxycholate and 0.1% SDS) supplemented with 0.2 mM sodium vanadate, 50 mM sodium fluoride, 0.1 mM PMSF and protease inhibitor cocktail (Roche). Supernatant was quantitated by the BCA protein assay kit (Pierce). Western blots were performed with antibodies specific for phospho-p38 (Cell signaling #9211), proteasome 20S α subunits (BIOMOL #8195), and α-tubulin (Sigma-Aldrich #9026).

### Oxidative stress resistance assays

Analyses of oxidative stress resistance were performed essentially as described [23]. To assay TBHP resistance, L3/L4 stage animals were fed with RNAi bacteria for three days, then transferred to plates that contained 9.125 mM TBHP (Sigma-Aldrich) in the agar and *E. coli* OP50 food. Fresh TBHP plates were prepared fresh two hours before transfer. Animals that bagged, crawled off the plates and exploded were censored. As resistance assays were performed by essentially the same method, using freshly prepared plates that contained 10 mM NaAsO_2_ (Sigma-Aldrich) in the agar and *E. coli* OP50 food.

### Lifespan analysis

Lifespan analyses were conducted at 20°C, with RNAi treatments performed only during adulthood. N2 animals were synchronized by timed egglay for 2–4 hours on plates seeded with control RNAi bacteria. Synchronized one-day-old adults were transferred to lifespan plates seeded with gene-specific or control RNAi bacteria. 2′ fluoro-5′ deoxyuridine (FUDR) was present (0.1 mg/ml) to prevent progeny development. The first day of adulthood was used as t = 0, with animals scored each day after the sixteenth day of adulthood. Those that crawled off the plate, exploded, or bagged were censored. JMP version 7, was used for statistical analyses, and *P* values were calculated using the log-rank method.

### *In vivo* proteasome activity assay

L2/L3 larvae were placed on RNAi feeding plates, then exposed to photoconversion after 72 h (muscle cell imaging) or 48 h (all others). Photoconversion and image analysis were performed as described [40]. Only gravid adults were imaged, and worms were maintained on RNAi plates between time points. *P* values were determined by Student's t-test (homoscedastic).

### Quantification and imaging of GFP reporters

For proteasome reporters, L2/L3 larvae were fed RNAi bacteria for 3 days, then normal-appearing worms that developed into gravid adults were analyzed. Animals were mounted on 5% agarose pads, immobilized in 1 mM levamisole and imaged with a Zeiss Axioplan 2 microscope. Confocal microscopy was used to generate z-stack projections for a representative subset of animals (LSM 510 Meta, 40× plan-neofluar objective, Zeiss, Germany; z-stacks with 0.5 µm interval). At least two stable transgenic lines for each proteasome reporter strain were examined. Fluorescent images were analyzed by the MCID system (Imaging Research) to measure the average fluorescence level of the entire worm, or particular regions. The average value of controls for each experiment was set as 100%, with values obtained in parallel from RNAi-treated worms converted to the relative fluorescence level. Data obtained from several experiments were pooled for statistical analysis.

For other reporters, an AxioVision (Zeiss) microscope was used to acquire imaging and fluorescence was scored by eye as Low, Medium, or High as described for each experiment.

Additional Materials and Methods are provided in Text S1.

## Supporting Information

Figure S1 Schematic of the translation elongation cycle. eEF1A is involved in delivering aminoacyl-tRNA to the empty A-site of the ribosome in the presence of GTP. eEF1B is a multi-subunit nucleotide exchange factor that partners with eEF1A, and enhances the recycling of eEF1A-GDP to eEF1A-GTP. eEF2 is a monomeric protein that translocates peptidyl tRNA to the P-site. After translocation, the peptidyl-tRNA is positioned in the ribosome P-site, and the next codon on the mRNA is made available for the next elongation cycle [49].
(TIF)

Figure S2 SKN-1 target gene induction in response to inhibition of translation elongation. (A) Diagram of the *gcs-1* promoter transgenes used in this study [24]. 1, 2, and 3 refer to SKN-1 binding sites. Mutation of site 3 abolishes most *skn-1*-dependent expression. (B) Expression of *gcs-1p*::GFP is dependent upon SKN-1 binding site 3, and p38 signaling through the MAPKK SEK-1. *gcs-1p*::GFP expression was scored as in Figure 1A, after RNAi against the indicated TEF. *P* values were derived from a chi^2^ test, and were all above 0.009. (C) SKN-1-dependence of endogenous target gene induction. In all qRT-PCR figures, ***P<0.001, **P<0.01, *P<0.05, N = not significant, and error bars indicate SEM. Endogenous *atf-5* or *haf-7* mRNA was detected by qRT-PCR in wild-type (N2) or *skn-1(zu135)* animals that had been fed with TEF RNAi bacteria, or treated with CHX for 18 hs at 15°C. A paired t test (two-tailed) was employed to compare wild-type (N2) and *skn-1(zu135)* animals. An unpaired t test (two-tailed) was used to compare TEF RNAi or CHX treatment vs the corresponding control in N2 animals. Compared to N2 control, all *P*<0.05. (D) Intestinal *gst-4p*::GFP expression is not robustly induced by TEF RNAi. Worms were scored for GFP expression after RNAi knockdown of the indicated TEFs or the TIF *ifg-1*, with examples of high and low scoring provided. “High” indicates that *gst-4p*::GFP was present at unambiguously high levels throughout most of the intestine, while “low” refers to animals in which readily detectable GFP signal was present only in the most anterior part of the intestine, and “medium” indicates an intermediate level of GFP signal. *P* values were derived from a chi^2^ test. *** *P*<0.0001, ** *P*<0.005, NS = Not Significant. (E) Examples of SKN-1::GFP accumulation in intestinal nuclei that scored as low, medium and high in Figure 1F. “Low” refers to animals in which GFP was barely detectable in nuclei throughout the intestine, “medium” indicates that GFP was present in the anterior and/or posterior intestinal nuclei, and “High” indicates that a strong GFP signal was present in most intestinal nuclei. Arrows indicate intestinal nuclei. (F) Relative *skn-1* mRNA levels after TEF RNAi or CHX treatment, measured by qRT-PCR. (G) The p38 MAPKK SEK-1 and MAPKKK NSY-1 are required for p38 MAPK activation in response to TEF RNAi. In *C. elegans*, canonical p38 signaling is blocked by mutation of either *sek-1* or *nsy-1* [29]. Representative experiments are shown in which lysates from control or RNAi worms were analyzed by Western blotting for p38 kinase phosphorylation (activation) as in Figure 1G. In all panels α-tubulin was the loading control. (H) p38 activation in response to knockdown of TIFs, assayed as in (G). In this and multiple other experiments only *ifg-1* RNAi increased levels of p38 phosphorylation comparably to TEF RNAi. This p38 activation required the p38 MAPKK *sek-1* (right panel).
(TIF)

Figure S3 Effects of TEF RNAi do not derive from a global induction of stress responses. (A, B) Heat shock genes are activated by proteasomal gene knockdown but not TEF RNAi. In (A), a transgenic reporter driven by the promoter for the heat-shock gene *hsp-70* (*hsp-70p::GFP*) [50] was robustly upregulated in the anterior and posterior intestine after proteasomal subunit gene RNAi (*rpn-2*, *rpn-4*), but not TEF RNAi. In (B), the GFP-fused promoter for the small heat shock protein gene *hsp-16.2* [51] was induced by proteasomal subunit RNAi but not TEF knockdown. “Low” indicates that GFP was undetectable throughout the animal, “medium” indicates that GFP was present in the middle intestinal nuclei, and “High” indicates that GFP signal was present in most intestinal nuclei. (C) RNAi against TEFs decreased fecundity. *P* values were derived from an unpaired t test (two-tailed). For each RNAi treatment *P*<0.0001 compared with corresponding control. (D) DAF-16::GFP does not accumulate in intestinal nuclei in response to TEF knockdown, in contrast to the effect of decreased germ cell proliferation. *P* values were derived from a chi^2^ test; *** *P*<0.0001, **P<0.005, here and in (E). (E) Expression of the DAF-16 target gene reporter *sod-3p*::GFP after TEF RNAi. *sod-3p*::GFP is robustly induced by knockdown of the insulin receptor DAF-2, or by inhibition of germ cell proliferation [38], but is only modestly affected by TEF RNAi. For control and TEF RNAi, “high” corresponds to a bright GFP signal being present throughout the hypodermis (in both the cytoplasm and nucleus) and posterior intestine, “medium” refers to modest GFP expression in the anterior and posterior intestine, and “low” indicates modest GFP expression in the posterior intestine only. For *daf-2* RNAi, a strong GFP signal was present throughout both the intestine and hypodermis. (F) Lifespan analysis of TEF RNAi worms, performed in parallel to Figure 3D. For control, *eef-1A.1 and eef-1G* RNAi treatments, composites of two biological replicates are shown. For *ifg-1* RNAi, a single experiment is shown. Statistics are provided in Table S5B.
(TIF)

Figure S4 Effects of proteosomal subunit and *skn-1* RNAi on proteasome gene expression and UPS activity *in vivo*. (A, B) Requirement for *skn-1* for the “bounce-back” response to proteasome gene RNAi. Confocal z-stack projection images are shown of representative 2-day-old adult worms that carry proteasome gene reporter transgenes, and were subjected to the indicated RNAi treatments. *RPN-11::GFP* is a translational fusion reporter, but *pbs-4p::GFP* includes only the *pbs-4* promoter region. For all worms in double RNAi experiments, z-stack projections through the intestine or body-wall muscle are shown. Dashed lines indicate boundaries of the intestine. Abbreviations: M, body-wall muscle; SIM, stomatointestinal muscle. Quantification and statistics are listed in Table S6. In all double RNAi experiments, RNAi and/or control bacteria were mixed at a 1∶1 volume ratio, with single RNAi treatments mixed with control. (C) Knockdown of proteasome subunits impairs intestinal UPS activity. Representative images of animals fed control (L4440), *pbs-5* (20S β-ring), *rpn-2* (19S non-ATPase) and *rpt-4* (19S ATPase) RNAi respectively. Bar: 20 µm. Note the difference in % UbG76V-Dendra2 fluorescence remaining after 9 hours. (D) SKN-1 does not contribute to UPS-mediated protein degradation in body-wall muscle cells. UbG76V-Dendra2 and control Dendra2 that were expressed specifically in body-wall muscle cells (from *Punc-54*) were imaged in control and *skn-1* RNAi animals at 24 hours after photoconversion. Bar: 20 µm. Depicted in the graphs: percentages of green and red fluorescence related to the initial value (t = 0) or point of photoconversion (t = C) respectively (± SEM). *P* = 0.1787 (Student's t-test). (E) SKN-1 is not required for UPS-mediated UbG76V-Dendra2 degradation in dopaminergic neurons, assayed at 9 hours after photoconversion. Representative experiment is shown. UbG76V-Dendra2 and control Dendra2 that were expressed specifically in dopaminergic neurons (from *Pdat-1*) were imaged in control and *skn-1* RNAi animals at 9 hours after photoconversion. Experiments were performed in the RNAi-sensitive strain *rrf-3 (pk1426)* to allow penetrance in neurons. Note that degradation slightly increased by *skn-1* RNAi. Bar: 5 µm. Numbers of animals and statistics for all experiments are listed in Table S7.
(TIF)

Figure S5 Importance of SKN-1 for proteasome function. (A) SKN-1 is required for total *C. elegans* proteasome activity, as measured by a proteasome in-gel activity assay. The left panels show fluorescent (top) and Coomassie-stained (bottom) images of a representative experiment in which the chymotrypsin-like activity of the proteasome was assayed. CP refers to the 20S proteasome core particle, and RP to the 19S regulatory particle. The 26S complexes designated as RP-CP and RP_2_-CP include RPs at one or both ends of the CP, respectively. The right panel shows the relative fold-change in normalized substrate fluorescence compared to control (set as 1). Results of four individual experiments are graphed, with error bars that correspond to SEM. (B) Knockdown of *skn-1* by RNAi feeding increases sensitivity to proteasome inhibition. A representative experiment (of three total) is shown in which one day-old adults were fed L4440 control (in blue) or *skn-1* RNAi (in red) bacteria for three days, exposed to the indicated concentration of the proteasome inhibitor MG132 in 1% DMSO for 24 hours, then scored for viability. N = approx. 50 in each of two wells, and error bars indicate SEM.
(TIF)

Figure S6 Distinct effects of TEF, TIF, and proteasome subunit RNAi on proteasome subunit and SKN-1 target gene expression. (A) Relative levels of endogenous proteasome subunit mRNAs after TEF or TIF RNAi. In contrast to results seen after proteasome subunit gene knockdown, in most cases RNAi against these translation factors modestly reduced proteasome gene expression. N = not significant, all other *P*<0.05. (B) Representative confocal images of the proteasome reporter *pas-5p::GFP* fed either control or *eef-2* RNAi. Note that GFP levels were reduced in the intestine in response to knockdown of *eef-2*. (C, D) Reduced levels of proteasome 20S α subunits after RNAi against TEFs or TIFs. Lysates from control or RNAi worms were Western blotted with an antibody against 20S proteasome α1, 2, 3, 5, 6 & 7 subunits [52]. For TEF RNAi (C), the nitrocellulose transfer membrane used was same as that used in Figure S2G. For TIF RNAi (D), the membrane was same as that used in Figure S2H. Representative experiments are shown. (E) p38 MAPK is not activated by RNAi against proteasome subunits, in contrast to effects of TEF RNAi. Western blot assay was performed as in Figure 1G, Figure S2G and S2H. (F) RNAi against proteasome subunits does not dramatically increase SKN-1 B/C::GFP accumulation in intestinal nuclei. Worms were scored as in Figure 1F. (G) Relative levels of endogenous proteasome (*rpt-3*, *rpn-12*, *pas-4* and *pbs-6*) and SKN-1 target (*gcs-1*, *gst-4* and *gst-10*) mRNAs after proteasome subunit (*rpn-2* or *rpt-4*) RNAi, assayed by qRT-PCR. These genes are not generally induced by TEF RNAi, except for *gcs-1*. *tba-1* (α-tubulin) was used for normalization, here and in (H). N = not significant, all other *P*<0.02. (H) SKN-1-dependent upregulation of *gst-4* and *gst-10* by proteasome subunit RNAi. Statistical analysis was performed as in Figure S2C. All *P*<0.05 compared to control, except where not significant is indicated by N.
(TIF)

Table S1 Mammalian and *C. elegans* TEFs. Identity (%) is from NCBI/basic BLAST/protein blast program. *Isoform chosen to run BLAST.
(DOCX)

Table S2 Effects of TEF RNAi on resistance of wild type worms to 9.125 mM TBHP. The third individual experiment described above is graphed in Figure 3A. JMP software was used for data analysis. Percentage change of mean survival time = (mean survival time of animals fed treatment RNAi - mean survival time of animals fed control RNAi)/mean survival time of animals fed control RNAi. 75^th^ percentiles refer to the time at which 75% population was dead. Wild type N2 animals were used in each RNAi experiment. No. RNAi animals indicates the number of observed deaths/total number of worms subjected to RNAi treatment. *P* values were calculated by log-rank.
(DOCX)

Table S3 *skn-1*-dependence of TBHP oxidative stress resistance. Three individual experiments are listed that were performed in parallel as in Figure 3B. The third individual experiment labeled with * is shown in Figure 3B. Data were analyzed as in Table S2.
(DOCX)

Table S4 Effects of TEF RNAi on resistance of wild type worms to 10 mM Arsenite. Individual experiments are listed that were performed as in Figure 3C. The first individual experiment is shown in Figure 3C. Data were analyzed as in Table S2.
(DOCX)

Table S5 Lifespan analysis of inhibition of translation. Corresponds to the data in Figure 3D and S3F, respectively. Data of each table was a composite of multiple individual experiments. Percentage increase in mean lifespan = (mean RNAi treatment adult lifespan- mean control adult lifespan)/mean control adult lifespan. 75^th^ and 25^th^ percentiles refer to the day at which 75% or 25% the population was dead. Wild type N2 animals were used for the experiments. N represents number of RNAi worms, number of observed deaths/total number of worms subjected to RNAi treatment. *P* values were calculated by log-rank.
(DOCX)

Table S6 Quantification and statistical analysis of proteasome subunit reporter expression. Fluorescence densities in whole worms or selected regions (intestine, head) were obtained from the measurement of fluorescent microscopic images by a computer-based MCID image analysis system. For each experiment, the average values of control worms (fed with L4440 RNAi bacteria) were set as 1, with values obtained from RNAi-treated worms in parallel converted to relative fluorescence levels. Data obtained from several experiments were pooled for t tests (two-tailed). The number of independent experiments was indicated as N. For each proteasome subunit reporter, two to three transgenic lines of worms were examined in each experiment. Data correspond to results shown in Figure 4B, 4C; Figure S4A, S4B and S6B.
(DOCX)

Table S7 Statistical analysis of *in vivo* UPS activity experiments. Number of individual experiments is shown in parentheses. Data correspond to results shown in Figure 5 and S4C–S4E.
(DOCX)

Table S8 Primer sequences. This table includes all primers used in qRT-PCR experiments.
(DOCX)

Text S1 Supplementary Materials and Methods.
(DOC)
