# Supplementary figures and images for: Specific SKN-1/Nrf Stress Responses to Perturbations in Translation Elongation and Proteasome Activity
Source: PLoS Genet. 2011 Jun 9;7(6):e1002119. doi: 10.1371/journal.pgen.1002119 (PMC3111486; doi:10.1371/journal.pgen.1002119)

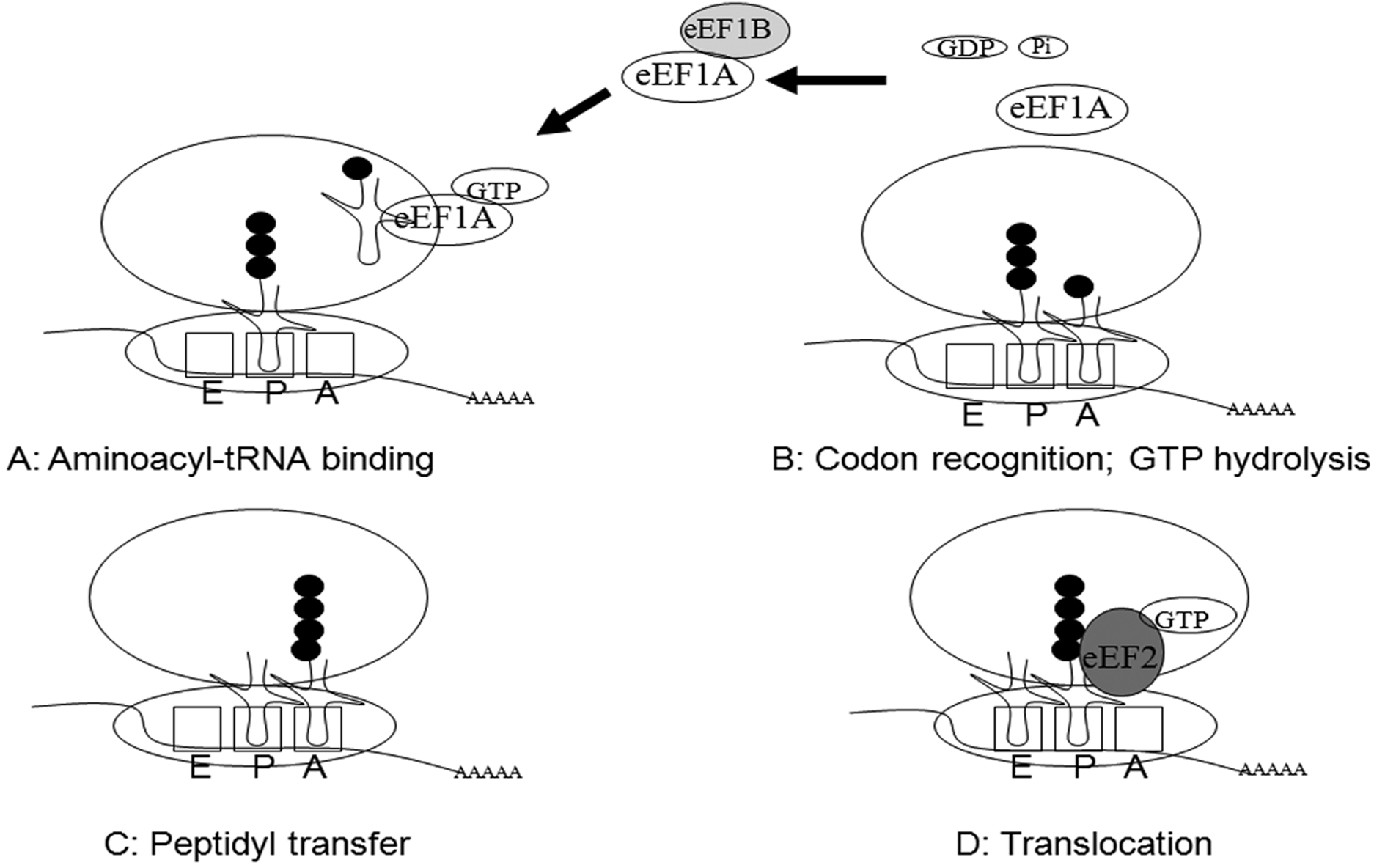

Supplement: Figure S1 — Schematic of the translation elongation cycle. eEF1A is involved in delivering aminoacyl-tRNA to the empty A-site of the ribosome in the presence of GTP. eEF1B is a multi-subunit nucleotide exchange factor that partners with eEF1A, and enhances the recycling of eEF1A-GDP to eEF1A-GTP. eEF2 is a monomeric protein that translocates peptidyl tRNA to the P-site. After translocation, the peptidyl-tRNA is positioned in the ribosome P-site, and the next codon on the mRNA is made available for the next elongation cycle [49]. (TIF) [file pgen.1002119.s001.tif]

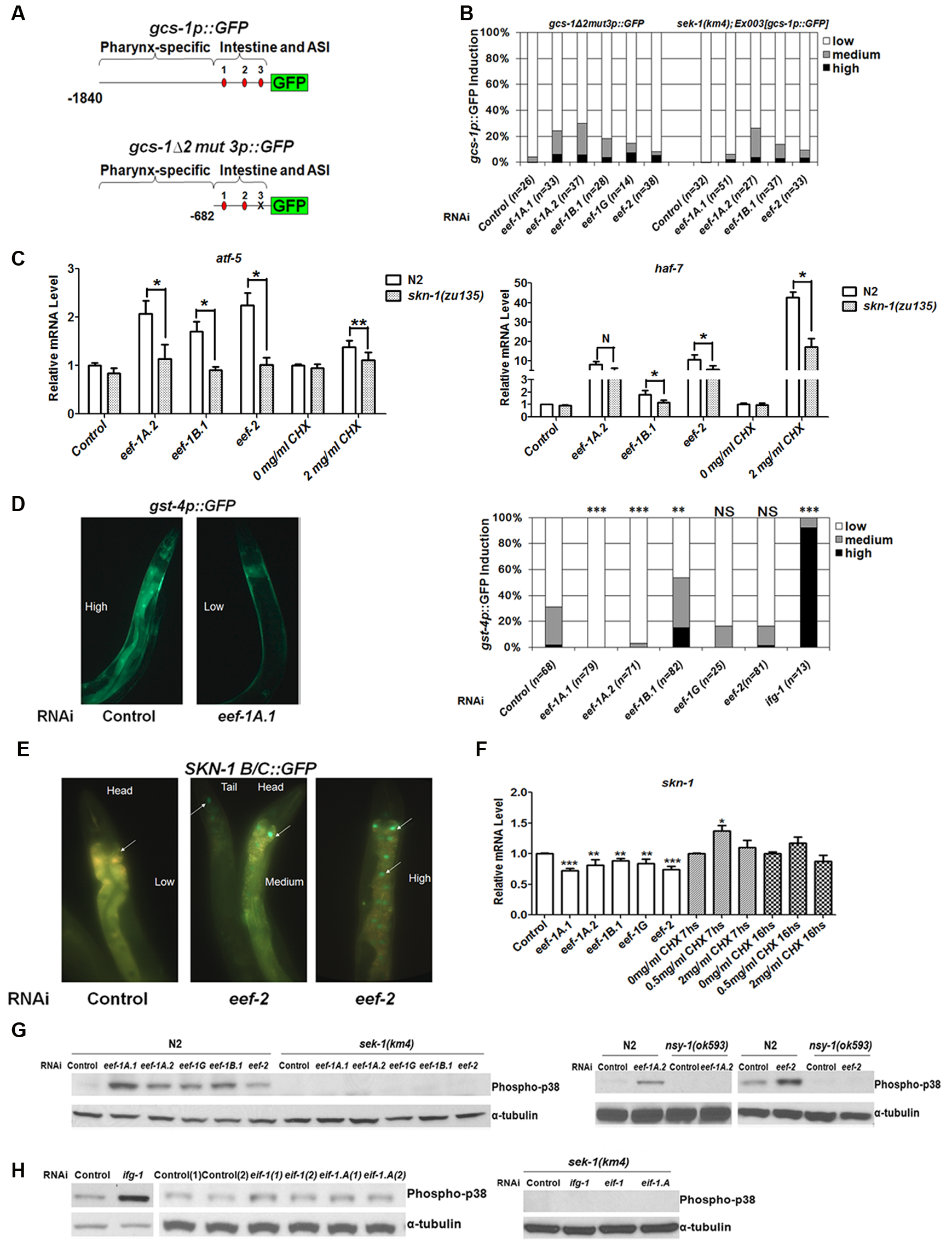

Supplement: Figure S2 — SKN-1 target gene induction in response to inhibition of translation elongation. (A) Diagram of the gcs-1 promoter transgenes used in this study [24]. 1, 2, and 3 refer to SKN-1 binding sites. Mutation of site 3 abolishes most skn-1-dependent expression. (B) Expression of gcs-1p::GFP is dependent upon SKN-1 binding site 3, and p38 signaling through the MAPKK SEK-1. gcs-1p::GFP expression was scored as in Figure 1A, after RNAi against the indicated TEF. P values were derived from a chi2 test, and were all above 0.009. (C) SKN-1-dependence of endogenous target gene induction. In all qRT-PCR figures, ***P<0.001, **P<0.01, *P<0.05, N = not significant, and error bars indicate SEM. Endogenous atf-5 or haf-7 mRNA was detected by qRT-PCR in wild-type (N2) or skn-1(zu135) animals that had been fed with TEF RNAi bacteria, or treated with CHX for 18 hs at 15°C. A paired t test (two-tailed) was employed to compare wild-type (N2) and skn-1(zu135) animals. An unpaired t test (two-tailed) was used to compare TEF RNAi or CHX treatment vs the corresponding control in N2 animals. Compared to N2 control, all P<0.05. (D) Intestinal gst-4p::GFP expression is not robustly induced by TEF RNAi. Worms were scored for GFP expression after RNAi knockdown of the indicated TEFs or the TIF ifg-1, with examples of high and low scoring provided. “High” indicates that gst-4p::GFP was present at unambiguously high levels throughout most of the intestine, while “low” refers to animals in which readily detectable GFP signal was present only in the most anterior part of the intestine, and “medium” indicates an intermediate level of GFP signal. P values were derived from a chi2 test. *** P<0.0001, ** P<0.005, NS = Not Significant. (E) Examples of SKN-1::GFP accumulation in intestinal nuclei that scored as low, medium and high in Figure 1F. “Low” refers to animals in which GFP was barely detectable in nuclei throughout the intestine, “medium” indicates that GFP was present in the anterior and/or posteri [file pgen.1002119.s002.tif]

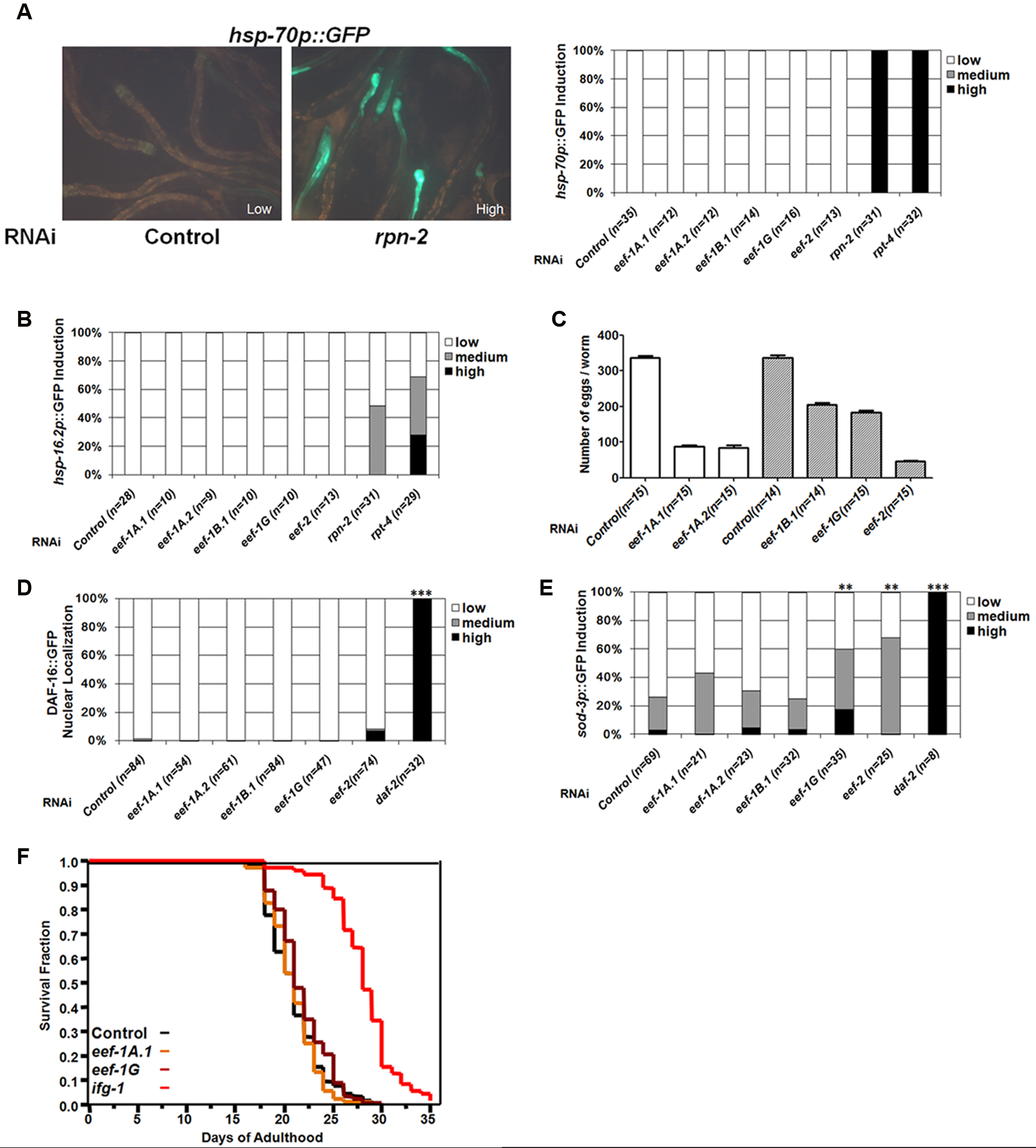

Supplement: Figure S3 — Effects of TEF RNAi do not derive from a global induction of stress responses. (A, B) Heat shock genes are activated by proteasomal gene knockdown but not TEF RNAi. In (A), a transgenic reporter driven by the promoter for the heat-shock gene hsp-70 (hsp-70p::GFP) [50] was robustly upregulated in the anterior and posterior intestine after proteasomal subunit gene RNAi (rpn-2, rpn-4), but not TEF RNAi. In (B), the GFP-fused promoter for the small heat shock protein gene hsp-16.2 [51] was induced by proteasomal subunit RNAi but not TEF knockdown. “Low” indicates that GFP was undetectable throughout the animal, “medium” indicates that GFP was present in the middle intestinal nuclei, and “High” indicates that GFP signal was present in most intestinal nuclei. (C) RNAi against TEFs decreased fecundity. P values were derived from an unpaired t test (two-tailed). For each RNAi treatment P<0.0001 compared with corresponding control. (D) DAF-16::GFP does not accumulate in intestinal nuclei in response to TEF knockdown, in contrast to the effect of decreased germ cell proliferation. P values were derived from a chi2 test; *** P<0.0001, **P<0.005, here and in (E). (E) Expression of the DAF-16 target gene reporter sod-3p::GFP after TEF RNAi. sod-3p::GFP is robustly induced by knockdown of the insulin receptor DAF-2, or by inhibition of germ cell proliferation [38], but is only modestly affected by TEF RNAi. For control and TEF RNAi, “high” corresponds to a bright GFP signal being present throughout the hypodermis (in both the cytoplasm and nucleus) and posterior intestine, “medium” refers to modest GFP expression in the anterior and posterior intestine, and “low” indicates modest GFP expression in the posterior intestine only. For daf-2 RNAi, a strong GFP signal was present throughout both the intestine and hypodermis. (F) Lifespan analysis of TEF RNAi worms, performed in parallel to Figure 3D. For control, eef-1A.1 and eef-1G RNAi treatments, composites of two biological replica [file pgen.1002119.s003.tif]

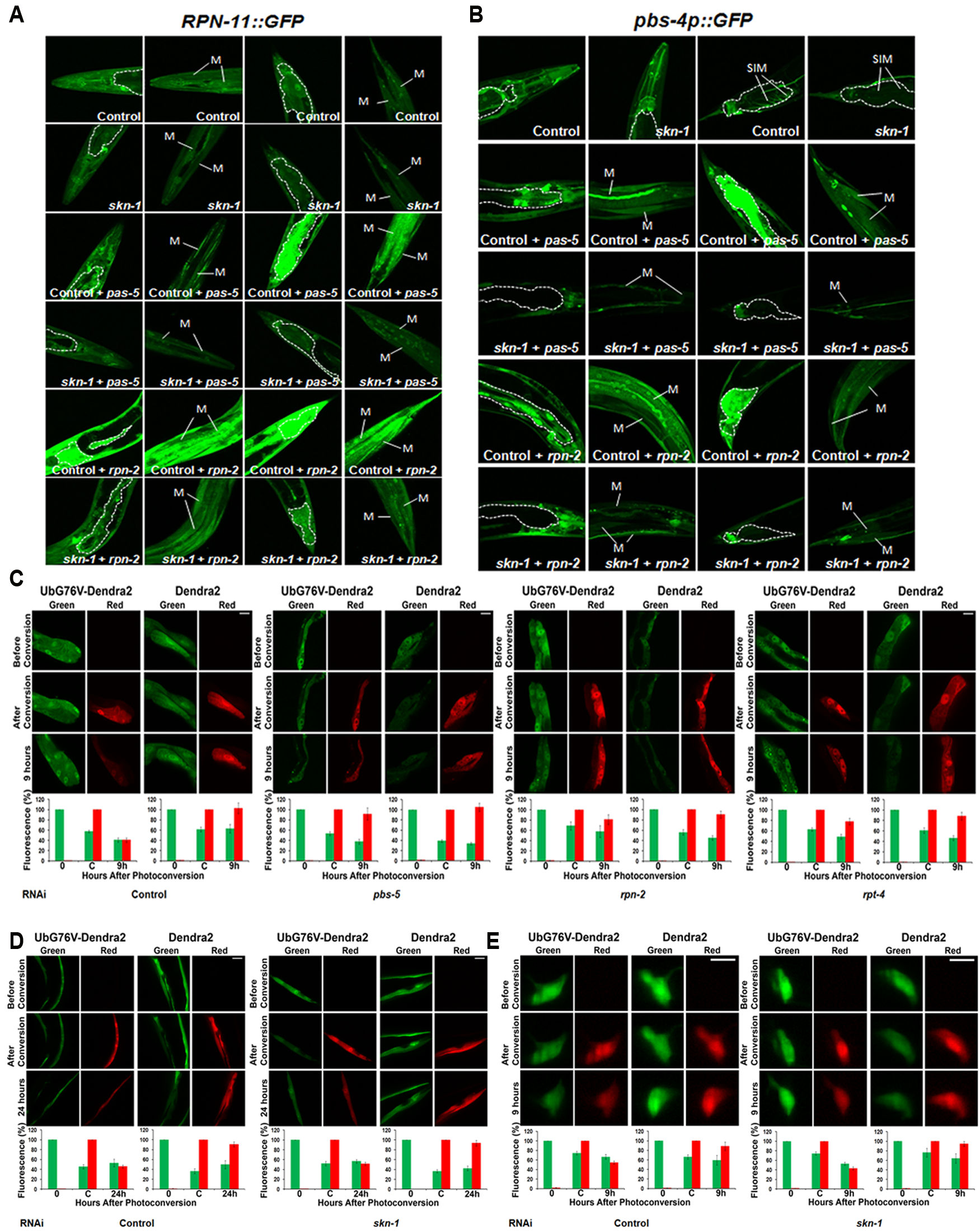

Supplement: Figure S4 — Effects of proteosomal subunit and skn-1 RNAi on proteasome gene expression and UPS activity in vivo. (A, B) Requirement for skn-1 for the “bounce-back” response to proteasome gene RNAi. Confocal z-stack projection images are shown of representative 2-day-old adult worms that carry proteasome gene reporter transgenes, and were subjected to the indicated RNAi treatments. RPN-11::GFP is a translational fusion reporter, but pbs-4p::GFP includes only the pbs-4 promoter region. For all worms in double RNAi experiments, z-stack projections through the intestine or body-wall muscle are shown. Dashed lines indicate boundaries of the intestine. Abbreviations: M, body-wall muscle; SIM, stomatointestinal muscle. Quantification and statistics are listed in Table S6. In all double RNAi experiments, RNAi and/or control bacteria were mixed at a 1∶1 volume ratio, with single RNAi treatments mixed with control. (C) Knockdown of proteasome subunits impairs intestinal UPS activity. Representative images of animals fed control (L4440), pbs-5 (20S β-ring), rpn-2 (19S non-ATPase) and rpt-4 (19S ATPase) RNAi respectively. Bar: 20 µm. Note the difference in % UbG76V-Dendra2 fluorescence remaining after 9 hours. (D) SKN-1 does not contribute to UPS-mediated protein degradation in body-wall muscle cells. UbG76V-Dendra2 and control Dendra2 that were expressed specifically in body-wall muscle cells (from Punc-54) were imaged in control and skn-1 RNAi animals at 24 hours after photoconversion. Bar: 20 µm. Depicted in the graphs: percentages of green and red fluorescence related to the initial value (t = 0) or point of photoconversion (t = C) respectively (± SEM). P = 0.1787 (Student's t-test). (E) SKN-1 is not required for UPS-mediated UbG76V-Dendra2 degradation in dopaminergic neurons, assayed at 9 hours after photoconversion. Representative experiment is shown. UbG76V-Dendra2 and control Dendra2 that were expressed specifically in dopaminergic neurons (from Pdat-1) were imaged in control and [file pgen.1002119.s004.tif]

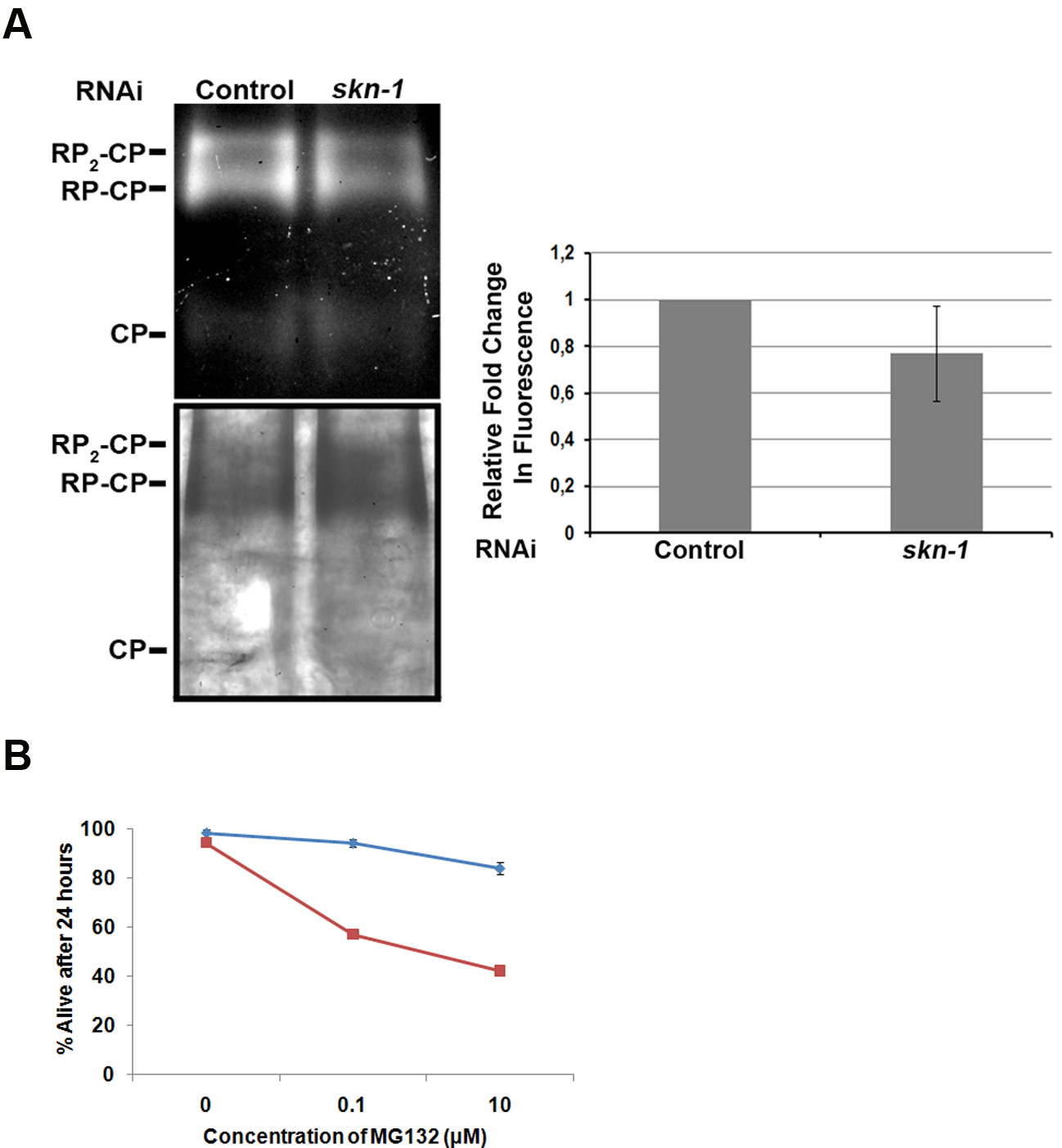

Supplement: Figure S5 — Importance of SKN-1 for proteasome function. (A) SKN-1 is required for total C. elegans proteasome activity, as measured by a proteasome in-gel activity assay. The left panels show fluorescent (top) and Coomassie-stained (bottom) images of a representative experiment in which the chymotrypsin-like activity of the proteasome was assayed. CP refers to the 20S proteasome core particle, and RP to the 19S regulatory particle. The 26S complexes designated as RP-CP and RP2-CP include RPs at one or both ends of the CP, respectively. The right panel shows the relative fold-change in normalized substrate fluorescence compared to control (set as 1). Results of four individual experiments are graphed, with error bars that correspond to SEM. (B) Knockdown of skn-1 by RNAi feeding increases sensitivity to proteasome inhibition. A representative experiment (of three total) is shown in which one day-old adults were fed L4440 control (in blue) or skn-1 RNAi (in red) bacteria for three days, exposed to the indicated concentration of the proteasome inhibitor MG132 in 1% DMSO for 24 hours, then scored for viability. N = approx. 50 in each of two wells, and error bars indicate SEM. (TIF) [file pgen.1002119.s005.tif]

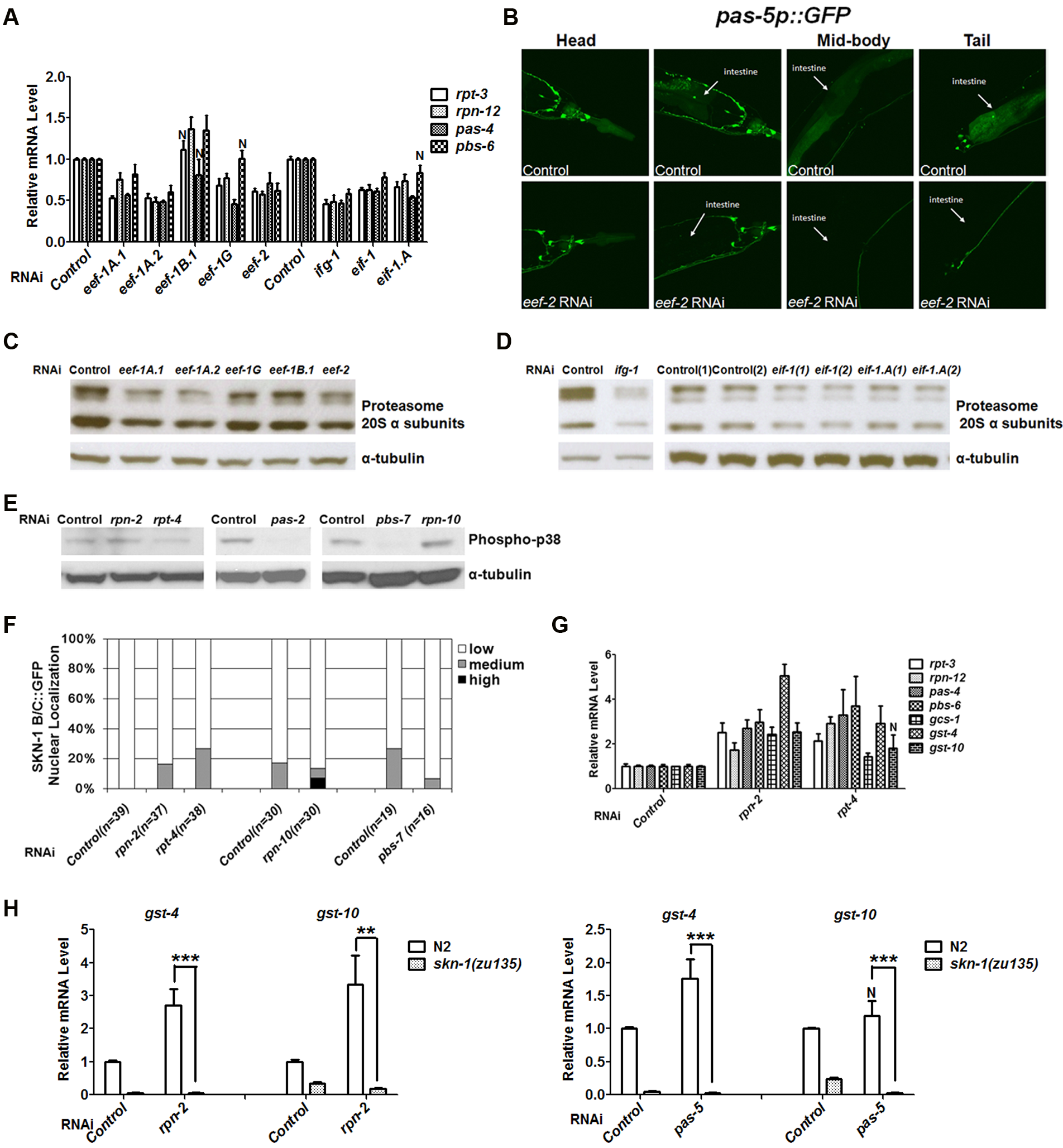

Supplement: Figure S6 — Distinct effects of TEF, TIF, and proteasome subunit RNAi on proteasome subunit and SKN-1 target gene expression. (A) Relative levels of endogenous proteasome subunit mRNAs after TEF or TIF RNAi. In contrast to results seen after proteasome subunit gene knockdown, in most cases RNAi against these translation factors modestly reduced proteasome gene expression. N = not significant, all other P<0.05. (B) Representative confocal images of the proteasome reporter pas-5p::GFP fed either control or eef-2 RNAi. Note that GFP levels were reduced in the intestine in response to knockdown of eef-2. (C, D) Reduced levels of proteasome 20S α subunits after RNAi against TEFs or TIFs. Lysates from control or RNAi worms were Western blotted with an antibody against 20S proteasome α1, 2, 3, 5, 6 & 7 subunits [52]. For TEF RNAi (C), the nitrocellulose transfer membrane used was same as that used in Figure S2G. For TIF RNAi (D), the membrane was same as that used in Figure S2H. Representative experiments are shown. (E) p38 MAPK is not activated by RNAi against proteasome subunits, in contrast to effects of TEF RNAi. Western blot assay was performed as in Figure 1G, Figure S2G and S2H. (F) RNAi against proteasome subunits does not dramatically increase SKN-1 B/C::GFP accumulation in intestinal nuclei. Worms were scored as in Figure 1F. (G) Relative levels of endogenous proteasome (rpt-3, rpn-12, pas-4 and pbs-6) and SKN-1 target (gcs-1, gst-4 and gst-10) mRNAs after proteasome subunit (rpn-2 or rpt-4) RNAi, assayed by qRT-PCR. These genes are not generally induced by TEF RNAi, except for gcs-1. tba-1 (α-tubulin) was used for normalization, here and in (H). N = not significant, all other P<0.02. (H) SKN-1-dependent upregulation of gst-4 and gst-10 by proteasome subunit RNAi. Statistical analysis was performed as in Figure S2C. All P<0.05 compared to control, except where not significant is indicated by N. (TIF) [file pgen.1002119.s006.tif]
