# Supplementary material for: Specific SKN-1/Nrf Stress Responses to Perturbations in Translation Elongation and Proteasome Activity
Source: PLoS Genet. 2011 Jun 9;7(6):e1002119. doi: 10.1371/journal.pgen.1002119 (PMC3111486; doi:10.1371/journal.pgen.1002119)
Supplement: Table S1 — Mammalian and C. elegans TEFs. Identity (%) is from NCBI/basic BLAST/protein blast program. *Isoform chosen to run BLAST. (DOCX) [file pgen.1002119.s007.docx]

**Table S1. Mammalian and *C. elegans* TEFs.**

| Mammal | *C. elegans* | *C. elegans*  (other name) | | Predicted identity | Sequence name | Number of amino acids | Identity to *C. briggsae* / *H. sapiens* homologs (%) |
| --- | --- | --- | --- | --- | --- | --- | --- |
| eEF1A | EEF-1A.1 | EFT-3 | Translation elongation factor 1A | | F31E3.5 | 463 | 98/84 |
| eEF1A | EEF-1A.2 | EFT-4 | Translation elongation factor 1A | | R03G5.1 | 463*,77,267,429 | 98/84 |
| eEF1B α/β | EEF-1B.1 |  | Translation elongation factor 1B α/β subunits | | F54H12.6 | 213 | 93/49 |
| eEF1B α/β | EEF-1B.2 |  | Translation elongation factor 1B α/β subunits | | Y41E3.10 | 263*,440 | 71/65 |
| eEF1B γ | EEF-1G |  | Glutathione S-transferase; Translation elongation factor 1B γ subunit | | F17C11.9 | 398*, 373, 365 | 95/46 |
| eEF2 | EEF-2 | EFT-2 | Translation elongation factor 2 | | F25H5.4 | 852 | 88/78 |
| eEF2 | EFTU-2 | EFT-1 | Translation elongation factor 2-like protein | | ZK328.2 | 974 | 92/37 |
