# Supplementary material for: Specific SKN-1/Nrf Stress Responses to Perturbations in Translation Elongation and Proteasome Activity
Source: PLoS Genet. 2011 Jun 9;7(6):e1002119. doi: 10.1371/journal.pgen.1002119 (PMC3111486; doi:10.1371/journal.pgen.1002119)
Supplement: Table S2 — Effects of TEF RNAi on resistance of wild type worms to 9.125 mM TBHP. The third individual experiment described above is graphed in Figure 3A. JMP software was used for data analysis. Percentage change of mean survival time = (mean survival time of animals fed treatment RNAi - mean survival time of animals fed control RNAi)/mean survival time of animals fed control RNAi. 75th percentiles refer to the time at which 75% population was dead. Wild type N2 animals were used in each RNAi experiment. No. RNAi animals indicates the number of observed deaths/total number of worms subjected to RNAi treatment. P values were calculated by log-rank. (DOCX) [file pgen.1002119.s008.docx]

**Table S2. Effects of TEF RNAi on resistance of wild type worms to 9.125mM TBHP.**

| RNAi treatment | RNAi mean survival (Hours±SEM) | 75^th^  percentile | No. RNAi animals | Percentage change of mean survival time | Percentage change of mean survival time (75^th^  Percentile) | *P* value against control |
| --- | --- | --- | --- | --- | --- | --- |
| Control | 24.19±0.44 | 26.5 | 63/66 |  |  |  |
| *skn-1* | 21.08±0.34 | 20 | 57/71 | -12.9 | -24.5 | < .0001 |
| *eef-1A.1* | 26.61±0.45 | 30 | 59/67 | 10.0 | 13.2 | 0.0006 |
| *eef-1A.2* | 26.72±0.49 | 30 | 74/85 | 10.5 | 13.2 | 0.0003 |
| *eef-1G* | 44.34±0.97 | 49 | 64/67 | 83.3 | 84.9 | < .0001 |
| Control | 43.92±0.81 | 45 | 65/75 |  |  |  |
| *skn-1* | 30.51±1.26 | 31.5 | 43/50 | -30.5 | -30.0 | < .0001 |
| *eef-1A.1* | 47.08±0.91 | 52.5 | 53/62 | 7.2 | 16.7 | 0.0028 |
| *eef-1A.2* | 47.82±0.61 | 48 | 55/63 | 8.9 | 6.7 | 0.0017 |
| *eef-1B.1* | 46.8±1.27 | 52.5 | 43/50 | 6.6 | 16.7 | 0.0012 |
| *eef-1G* | 57.67±1.23 | 69 | 56/62 | 31.3 | 53.3 | < .0001 |
| *eef-2* | 52.98±1.17 | 60 | 58/73 | 20.6 | 33.3 | < .0001 |
| Control | 37.59±0.88 | 39.5 | 45/45 |  |  |  |
| *skn-1* | 30.54±0.97 | 36 | 38/45 | -18.8 | -8.9 | < .0001 |
| *eef-1A.1* | 58.02±1.56 | 62.5 | 28/30 | 54.3 | 58.2 | < .0001 |
| *eef-1A.2* | 52.2±1.52 | 62.5 | 42/45 | 39.7 | 58.2 | < .0001 |
| *eef-1B.1* | 42.65±1.26 | 44 | 43/45 | 13.5 | 11.4 | 0.0036 |
| *eef-1G* | 63.64±2.09 | 74 | 29/30 | 69.3 | 87.3 | < .0001 |
| *eef-2* | 54.93±1.6 | 62.5 | 44/45 | 46.1 | 58.2 | < .0001 |
| Control | 37.8±1.34 | 44 | 51/60 |  |  |  |
| *eef-1A.2* | 60.43±1.42 | 66 | 54/60 | 59.9 | 50.0 | < .0001 |
| *eef-1B.1* | 44.51±1.44 | 48 | 38/40 | 17.8 | 9.1 | 0.0045 |
| *eef-2* | 61.56±1.09 | 62.5 | 49/60 | 62.9 | 42.0 | < .0001 |
