# Supplementary material for: Specific SKN-1/Nrf Stress Responses to Perturbations in Translation Elongation and Proteasome Activity
Source: PLoS Genet. 2011 Jun 9;7(6):e1002119. doi: 10.1371/journal.pgen.1002119 (PMC3111486; doi:10.1371/journal.pgen.1002119)
Supplement: Table S3 — skn-1-dependence of TBHP oxidative stress resistance. Three individual experiments are listed that were performed in parallel as in Figure 3B. The third individual experiment labeled with * is shown in Figure 3B. Data were analyzed as in Table S2. (DOCX) [file pgen.1002119.s009.docx]

**Table S3. *skn-1*-dependence of TBHP oxidative stress resistance.**

| Strain | RNAi treatment | RNAi mean survival (Hours±SEM) | 75^th^  percentile | No. RNAi animals | Percentage change of mean survival time | Percentage change of mean survival time (75^th^  Percentile) | *P* value against control |
| --- | --- | --- | --- | --- | --- | --- | --- |
| N2 | Control | 30.77±1.07 | 33.5 | 37/45 |  |  |  |
|  | Control | 45.83±2.01 | 51 | 30/30 |  |  |  |
|  | Control* | 36.8±1.65 | 44 | 35/45 |  |  |  |
|  | *eef-1G* | 40.48±1.04 | 45.5 | 42/45 | 31.6 | 35.8 | < .0001 |
|  | *eef-1G* | 59.07±1.77 | 67 | 30/30 | 28.9 | 31.4 | < .0001 |
|  | *eef-1G** | 57.2±1.56 | 63 | 41/45 | 55.4 | 43.2 | < .0001 |
|  | *eef-2* | 47.19±0.79 | 49.5 | 36/45 | 53.4 | 47.8 | < .0001 |
|  | *eef-2* | 57.51±1.4 | 67 | 45/45 | 25.5 | 31.4 | < .0001 |
|  | *eef-2** | 47.97±1.18 | 52 | 38/45 | 30.4 | 18.2 | < .0001 |
| *skn-1(zu135)* | Control | 20.91±0.38 | 20.5 | 45/45 |  |  |  |
|  | Control | 21.28±0.55 | 22 | 41/45 |  |  |  |
|  | Control* | 18.76±0.47 | 20 | 45/45 |  |  |  |
|  | *eef-1G* | 20.86±0.48 | 20.5 | 44/45 | -0.2 | 0 | 0.8659 |
|  | *eef-1G* | 25.26±0.92 | 26.5 | 45/45 | 18.7 | 20.5 | 0.0003 |
|  | *eef-1G** | 21.69±0.56 | 24 | 45/45 | 15.6 | 20 | 0.0003 |
|  | *eef-2* | 23.48±0.92 | 24.5 | 44/45 | 12.3 | 19.5 | 0.0011 |
|  | *eef-2* | 20.55±0.56 | 22 | 38/45 | -3.4 | 0 | 0.375 |
|  | *eef-2** | 21.64±0.46 | 24 | 44/45 | 15.4 | 20 | 0.0002 |
