# Supplementary material for: Specific SKN-1/Nrf Stress Responses to Perturbations in Translation Elongation and Proteasome Activity
Source: PLoS Genet. 2011 Jun 9;7(6):e1002119. doi: 10.1371/journal.pgen.1002119 (PMC3111486; doi:10.1371/journal.pgen.1002119)
Supplement: Table S4 — Effects of TEF RNAi on resistance of wild type worms to 10 mM Arsenite. Individual experiments are listed that were performed as in Figure 3C. The first individual experiment is shown in Figure 3C. Data were analyzed as in Table S2. (DOCX) [file pgen.1002119.s010.docx]

**Table S4.** **Effects of TEF RNAi on resistance of wild type worms to 10mM Arsenite.**

| RNAi treatment | RNAi mean survival (Hours±SEM) | 75^th^  percentile | No. RNAi animals | Percentage change of mean survival time | Percentage change of mean survival time (75^th^  Percentile) | *P* value against control |
| --- | --- | --- | --- | --- | --- | --- |
| Control | 14.64±1.1 | 18.5 | 65/75 |  |  |  |
| *skn-1* | 10.97±0.62 | 10.5 | 55/75 | -25.1 | -43.2 | 0.0044 |
| *eef-1A.1* | 26.11±1.53 | 34.5 | 46/61 | 78.3 | 86.5 | < .0001 |
| *eef-1A.2* | 23.23±1.61 | 34.5 | 54/75 | 58.7 | 86.5 | < .0001 |
| *eef-1B.1* | 19.77±1.3 | 30.5 | 59/74 | 35.0 | 64.9 | 0.0007 |
| *eef-2* | 22.91±1.41 | 30.5 | 50/75 | 56.5 | 64.9 | < .0001 |
| Control | 17.48±0.83 | 21 | 57/75 |  |  |  |
| *skn-1* | 15.11±0.71 | 21 | 61/75 | -13.6 | 0.0 | 0.0134 |
| *eef-1A.1* | 23.77±1.18 | 32.5 | 53/75 | 36.0 | 54.8 | < .0001 |
| *eef-1A.2* | 20.64±1.37 | 28.5 | 44/66 | 18.1 | 35.7 | 0.007 |
| *eef-1B.1* | 19.22±1.21 | 25 | 51/69 | 10.0 | 19.0 | 0.0197 |
| *eef-1G* | 16.64±1.25 | 25 | 59/75 | -4.8 | 19.0 | 0.4523 |
| *eef-2* | 20.08±1.11 | 28.5 | 60/75 | 14.9 | 35.7 | 0.0047 |
| Control | 20.59±0.9 | 22.5 | 50/75 |  |  |  |
| *skn-1* | 18.71±0.52 | 22.5 | 59/75 | -9.1 | 0.0 | 0.0016 |
| *eef-1A.1* | 19.7±0.74 | 22.5 | 54/75 | -4.3 | 0.0 | 0.2245 |
| *eef-1A.2* | 20.75±0.85 | 26.5 | 52/75 | 0.8 | 17.8 | 0.9577 |
| *eef-1B.1* | 23.4±1.27 | 30.5 | 61/75 | 13.6 | 35.6 | 0.0011 |
| *eef-1G* | 24.11±1.41 | 30.5 | 49/75 | 17.1 | 35.6 | 0.0001 |
| *eef-2* | 22.44±1.06 | 26.5 | 52/75 | 9.0 | 17.8 | 0.0732 |
