# Supplementary material for: Specific SKN-1/Nrf Stress Responses to Perturbations in Translation Elongation and Proteasome Activity
Source: PLoS Genet. 2011 Jun 9;7(6):e1002119. doi: 10.1371/journal.pgen.1002119 (PMC3111486; doi:10.1371/journal.pgen.1002119)
Supplement: Table S5 — Lifespan analysis of inhibition of translation. Corresponds to the data in Figure 3D and S3F, respectively. Data of each table was a composite of multiple individual experiments. Percentage increase in mean lifespan = (mean RNAi treatment adult lifespan- mean control adult lifespan)/mean control adult lifespan. 75th and 25th percentiles refer to the day at which 75% or 25% the population was dead. Wild type N2 animals were used for the experiments. N represents number of RNAi worms, number of observed deaths/total number of worms subjected to RNAi treatment. P values were calculated by log-rank. (DOCX) [file pgen.1002119.s011.docx]

**Table S5. Lifespan analysis of inhibition of translation.**

A

| Strain  (RNAi) | Mean adult lifespan±SEM | Percentage increase in mean lifespan (%) | 75^th^  percentile | 25^th^  percentile | *P* value against control | N | No. of Exp. |
| --- | --- | --- | --- | --- | --- | --- | --- |
| Control | 21.39±0.24 |  | 23 | 19 |  | 112/150 | 3 |
| *ifg-1* | 26.73 ±0.25 | 25 | 29 | 25 | < . 0001 | 178/207 | 3 |
| *eef-2* | 22.82 ±0.25 | 7 | 25 | 21 | 0. 0002 | 108/150 | 3 |
| *eef-1A.2* | 22.67 ±0.22 | 6 | 24 | 21 | 0. 0004 | 145/195 | 3 |
| *eef-1B.1* | 23.43 ±0.24 | 10 | 25 | 22 | < . 0001 | 123/161 | 3 |

B

| Strain  (RNAi) | Mean adult lifespan±SEM | Percentage increase in mean lifespan (%) | 75^th^  percentile | 25^th^  percentile | *P* value against control | N | No. of Exp. |
| --- | --- | --- | --- | --- | --- | --- | --- |
| Control | 20.97±0.32 |  | 23 | 19 |  | 70/100 | 2 |
| *ifg-1* | 28.16±0.39 | 34 | 30 | 26 | < . 0001 | 69/77 | 1 |
| *eef-1A.1* | 20.96±0.24 | 0 | 23 | 19 | 0.8636 | 93/115 | 2 |
| *eef-1G* | 21.80±0.23 | 4 | 24 | 20 | 0.0571 | 130/162 | 2 |
