# Supplementary material for: Specific SKN-1/Nrf Stress Responses to Perturbations in Translation Elongation and Proteasome Activity
Source: PLoS Genet. 2011 Jun 9;7(6):e1002119. doi: 10.1371/journal.pgen.1002119 (PMC3111486; doi:10.1371/journal.pgen.1002119)
Supplement: Table S6 — Quantification and statistical analysis of proteasome subunit reporter expression. Fluorescence densities in whole worms or selected regions (intestine, head) were obtained from the measurement of fluorescent microscopic images by a computer-based MCID image analysis system. For each experiment, the average values of control worms (fed with L4440 RNAi bacteria) were set as 1, with values obtained from RNAi-treated worms in parallel converted to relative fluorescence levels. Data obtained from several experiments were pooled for t tests (two-tailed). The number of independent experiments was indicated as N. For each proteasome subunit reporter, two to three transgenic lines of worms were examined in each experiment. Data correspond to results shown in Figure 4B, 4C; Figure S4A, S4B and S6B. (DOCX) [file pgen.1002119.s012.docx]

**Table S6. Quantification and statistical analysis of proteasome subunit reporter expression.**

| Strain  (region) | RNAi treatment | Mean | Numbers of animals and independent experiments (N) | *P* value against control | *P* value against control+proteasome subunit RNAi |
| --- | --- | --- | --- | --- | --- |
| *pas-5p::GFP* | Control | 1 | 27 |  |  |
| (whole worm) | *skn-1* | 0.865 | 9 (N = 2) | 0.3550 |  |
|  | *eef-2* | 0.697 | 16 (N = 1) | 0.0041 |  |
|  | *eef-1A.1* | 1.110 | 18 (N = 1) | 0.4405 |  |
|  | *pas-5* | 4.318 | 24 (N = 2) | 0.0034 |  |
|  | *rpn-2* | 4.558 | 11 (N = 2) | 1.71E-11 |  |
|  | *pbs-4* | 4.365 | 7 (N = 1) | 2.48E-06 |  |
| *pas-5p::GFP* | Control | 1 | 27 |  |  |
| (intestine) | *skn-1* | 0.640 | 9 (N = 2) | 0.0258 |  |
|  | *eef-2* | 0.463 | 16 (N = 1) | 6.04E-04 |  |
|  | *eef-1A.1* | 1.014 | 18 (N = 1) | 0.9489 |  |
|  | *pas-5* | 4.786 | 24 (N = 2) | 2.40E-11 |  |
|  | *rpn-2* | 4.470 | 11 (N = 2) | 1.08E-09 |  |
|  | *pbs-4* | 3.856 | 7 (N = 1) | 2.84E-08 |  |
| *pas-5p::GFP* | Control | 1 | 27 |  |  |
| (head) | *skn-1* | 1.055 | 9 (N = 2) | 0.5379 |  |
|  | *eef-2* | 0.967 | 16 (N = 1) | 0.6797 |  |
|  | *eef-1A.1* | 1.065 | 18 (N = 1) | 0.4561 |  |
|  | *pas-5* | 1.711 | 24 (N = 2) | 3.41E-04 |  |
|  | *rpn-2* | 1.740 | 11 (N = 2) | 0.0094 |  |
|  | *pbs-4* | 2.272 | 7 (N = 1) | 0.0088 |  |
| *pbs-4p::GFP* | Control | 1 | 38 |  |  |
| (whole worm) | *skn-1* | 0.923 | 27 (N = 2) | 0.3585 |  |
|  | *eef-2* | 1.000 | 17 (N = 1) | 0.9960 |  |
|  | *eef-1A.1* | 1.014 | 13 (N = 1) | 0.8897 |  |
|  | *pas-5* | 2.862 | 22 (N = 2) | 7.13E-15 |  |
|  | *rpn-2* | 2.715 | 15 ( N = 2) | 1.08E-10 |  |
| *pbs-4p::GFP* | Control | 1 | 38 |  |  |
| (intestine) | *skn-1* | 0.856 | 27 (N = 2) | 0.1432 |  |
|  | *eef-2* | 0.883 | 17 (N = 1) | 0.0618 |  |
|  | *eef-1A.1* | 0.878 | 13 (N = 1) | 0.1869 |  |
|  | *pas-5* | 2.421 | 22 (N = 2) | 2.28E-07 |  |
|  | *rpn-2* | 2.268 | 15 ( N = 2) | 2.05E-23 |  |
| *pbs-4p::GFP* | Control | 1 | 38 |  |  |
| (head) | *skn-1* | 1.004 | 27 (N = 2) | 0.9560 |  |
|  | *eef-2* | 0.954 | 17 (N = 1) | 0.3475 |  |
|  | *eef-1A.1* | 1.036 | 13 (N = 1) | 0.5177 |  |
|  | *pas-5* | 1.616 | 22 (N = 2) | 0.0290 |  |
|  | *rpn-2* | 1.436 | 15 ( N = 2) | 3.27E-04 |  |
| *pbs-4p::GFP* | Control+pas-5 | 1 | 22 |  |  |
| (whole worm) | *skn-1+pas-5* | 0.298 | 26 (N = 2) |  | 5.29E-22 |
| *pbs-4p::GFP* | Control*+pas-5* | 1 | 22 |  |  |
| (intestine) | *skn-1+pas-5* | 0.266 | 26 (N = 2) |  | 1.25E-28 |
| *pbs-4p::GFP* | Control*+pas-5* | 1 | 22 |  |  |
| (head) | *skn-1+pas-5* | 0.593 | 26 (N = 2) |  | 6.29E-12 |
| *pbs-4p::GFP* | Control*+rpn-2* | 1 | 24 |  |  |
| (whole worm) | *skn-1+ rpn-2* | 0.295 | 23 (N = 2) |  | 2.02E-18 |
| *pbs-4p::GFP* | Control*+ rpn-2* | 1 | 24 |  |  |
| (intestine) | *skn-1+ rpn-2* | 0.238 | 23 (N = 2) |  | 1.50E-24 |
| *pbs-4p::GFP* | Control*+ rpn-2* | 1 | 24 |  |  |
| (head) | *skn-1+ rpn-2* | 0.536 | 23 (N = 2) |  | 4.49E-15 |
| *rpn-11p::GFP* | Control | 1 | 32 |  |  |
| (whole worm) | *skn-1* | 0.735 | 16 (N = 2) | 1.39E-06 |  |
|  | *eef-2* | 0.930 | 14 (N = 1) | 0.5082 |  |
|  | *eef-1A.1* | 0.978 | 14 (N = 1) | 0.8092 |  |
|  | *pas-5* | 1.667 | 15 (N = 2) | 1.34E-04 |  |
|  | *rpn-2* | 1.964 | 17 (N = 2) | 3.86E-16 |  |
| *rpn-11p::GFP* | Control | 1 | 32 |  |  |
| (intestine) | *skn-1* | 0.717 | 16 (N = 2) | 3.40E-08 |  |
|  | *eef-2* | 0.861 | 14 (N = 1) | 0.1018 |  |
|  | *eef-1A.1* | 0.968 | 14 (N = 1) | 0.6811 |  |
|  | *pas-5* | 1.886 | 15 (N = 2) | 1.07E-05 |  |
|  | *rpn-2* | 1.939 | 17 (N = 2) | 2.39E-21 |  |
| *rpn-11p::GFP* | Control | 1 | 32 |  |  |
| (head) | *skn-1* | 0.904 | 16 (N = 2) | 0.2117 |  |
|  | *eef-2* | 1.152 | 14 (N = 1) | 0.3283 |  |
|  | *eef-1A.1* | 1.041 | 14 (N = 1) | 0.7113 |  |
|  | *pas-5* | 1.508 | 15 (N = 2) | 0.0121 |  |
|  | *rpn-2* | 1.611 | 17 (N = 2) | 3.81E-05 |  |
| *rpn-11p::GFP* | Control*+pas-5* | 1 | 24 |  |  |
| (whole worm) | *skn-1+pas-5* | 0.552 | 18 (N = 2) |  | 5.46E-13 |
| *rpn-11p::GFP* | Control*+pas-5* | 1 | 24 |  |  |
| (intestine) | *skn-1+pas-5* | 0.535 | 18 (N = 2) |  | 1.46E-10 |
| *rpn-11p::GFP* | Control*+pas-5* | 1 | 24 |  |  |
| (head) | *skn-1+pas-5* | 0.710 | 18 (N = 2) |  | 8.28E-07 |
| *rpn-11p::GFP* | Control*+rpn-2* | 1 | 18 |  |  |
| (whole worm) | *skn-1+ rpn-2* | 0.478 | 14 (N = 2) |  | 2.59E-14 |
| *rpn-11p::GFP* | Control*+ rpn-2* | 1 | 18 |  |  |
| (intestine) | *skn-1+ rpn-2* | 0.439 | 14 (N = 2) |  | 5.99E-18 |
| *rpn-11p::GFP* | Control*+ rpn-2* | 1 | 18 |  |  |
| (head) | *skn-1+ rpn-2* | 0.597 | 14 (N = 2) |  | 6.89E-10 |
| *rpt-5p::GFP* | Control | 1 | 40 |  |  |
| (whole worm) | *skn-1* | 0.826 | 14 (N = 2) | 0.1138 |  |
|  | *eef-2* | 0.952 | 18 (N = 1) | 0.7080 |  |
|  | *eef-1A.1* | 0.973 | 20 (N = 1) | 0.8435 |  |
|  | *pas-5* | 2.791 | 37 (N = 2) | 1.12E-10 |  |
|  | *rpn-2* | 3.145 | 29 (N = 2) | 1.03E-09 |  |
|  | *pbs-4* | 2.544 | 26 (N = 2) | 7.91E-09 |  |
| *rpt-5p::GFP* | Control | 1 | 40 |  |  |
| (intestine) | *skn-1* | 0.769 | 14 (N = 2) | 0.0235 |  |
|  | *eef-2* | 0.961 | 18 (N = 1) | 0.7738 |  |
|  | *eef-1A.1* | 0.901 | 20 (N = 1) | 0.4267 |  |
|  | *pas-5* | 5.460 | 37 (N = 2) | 4.13E-18 |  |
|  | *rpn-2* | 6.019 | 29 (N = 2) | 1.50E-14 |  |
|  | *pbs-4* | 5.650 | 26 (N = 2) | 1.47E-18 |  |
| *rpt-5p::GFP* | Control | 1 | 40 |  |  |
| (head) | *skn-1* | 0.968 | 14 (N = 2) | 0.7839 |  |
|  | *eef-2* | 1.012 | 18 (N = 1) | 0.9066 |  |
|  | *eef-1A.1* | 0.930 | 20 (N = 1) | 0.5585 |  |
|  | *pas-5* | 1.547 | 37 (N = 2) | 1.33E-05 |  |
|  | *rpn-2* | 1.786 | 29 (N = 2) | 2.00E-07 |  |
|  | *pbs-4* | 1.537 | 26 (N = 2) | 2.25E-04 |  |
| *rpt-5p::GFP* | Control | 1 | 40 |  |  |
| (whole worm) | Control*+pas-5* | 2.791 | 37 (N = 2) | 1.12E-10 |  |
|  | *skn-1+pas-5* | 1.049 | 18 (N = 2) | 0.7738 | 5.61E-09 |
| *rpt-5p::GFP* | Control | 1 | 40 |  |  |
| (intestine) | Control*+pas-5* | 5.460 | 37 (N = 2) | 4.13E-18 |  |
|  | *skn-1+pas-5* | 0.859 | 18 (N = 2) | 0.3229 | 3.85E-19 |
| *rpt-5p::GFP* | Control | 1 | 40 |  |  |
| (head) | Control*+pas-5* | 1.547 | 37 (N = 2) | 1.33E-05 |  |
|  | *skn-1+pas-5* | 1.003 | 18 (N = 2) | 0.9857 | 0.0013 |
| *rpt-5p::GFP* | Control | 1 | 40 |  |  |
| (whole worm) | Control*+rpn-2* | 3.145 | 29 (N = 2) | 1.03E-09 |  |
|  | *skn-1+ rpn-2* | 1.211 | 17 (N = 2) | 0.1587 | 51.70E-08 |
| *rpt-5p::GFP* | Control | 1 | 40 |  |  |
| (intestine) | Control*+ rpn-2* | 6.019 | 29 (N = 2) | 1.50E-14 |  |
|  | *skn-1+ rpn-2* | 1.150 | 17 (N = 2) | 0.2523 | 1.77E-14 |
| *rpt-5p::GFP* | *Control* | 1 | 40 |  |  |
| (head) | *Control+ rpn-2* | 1.786 | 29 (N = 2) | 2.00E-07 |  |
|  | *skn-1+ rpn-2* | 1.295 | 17 (N = 2) | 0.0296 | 0.0027 |
| *rpn-2p::GFP* | Control | 1 | 8 |  |  |
| (whole worm) | *skn-1* | 0.941 | 10 (N = 2) | 0.4293 |  |
|  | *pas-5* | 3.067 | 9 (N = 2) | 0.0079 |  |
| *rpn-2p::GFP* | Control | 1 | 8 |  |  |
| (intestine) | *skn-1* | 0.961 | 10 (N = 2) | 0.4602 |  |
|  | *pas-5* | 4.719 | 9 (N = 2) | 0.0004 |  |
| *rpn-2p::GFP* | Control | 1 | 8 |  |  |
| (head) | *skn-1* | 0.945 | 10 (N = 2) | 0.4691 |  |
|  | *pas-5* | 1.600 | 9 (N = 2) | 0.0686 |  |
| *RPN-11::GFP* | Control | 1 | 11 |  |  |
| (whole worm) | *skn-1* | 0.822 | 6 (N = 1) | 0.0477 |  |
| *RPN-11::GFP* | Control | 1 | 11 |  |  |
| (intestine) | *skn-1* | 0.837 | 6 (N = 1) | 0.0578 |  |
| *RPN-11::GFP* | Control | 1 | 11 |  |  |
| (head) | *skn-1* | 0.857 | 6 (N = 1) | 0.2724 |  |
| *RPN-11::GFP* | Control | 1 | 18 |  |  |
| (whole worm) | Control+*pas-5* | 1.818 | 12 (N = 2) | 5.50E-14 |  |
|  | *skn-1+pas-5* | 1.048 | 9 (N = 2) | 0.469 | 1.10E-08 |
|  | Control*+rpn-2* | 2.290 | 22 (N = 2) | 2.37E-08 |  |
|  | *skn-1+ rpn-2* | 1.073 | 11 (N = 2) | 0.218 | 5.54E-08 |
| *RPN-11::GFP* | Control | 1 | 18 |  |  |
| (intestine) | Control+*pas-5* | 1.824 | 12 (N = 2) | 1.36E-18 |  |
|  | *skn-1+pas-5* | 1.010 | 9 (N = 2) | 0.892 | 1.55E-07 |
|  | Control*+rpn-2* | 2.331 | 22 (N = 2) | 1.51E-07 |  |
|  | *skn-1+ rpn-2* | 1.144 | 11 (N = 2) | 0.010 | 8.96E-07 |
| *RPN-11::GFP* | Control | 1 | 18 |  |  |
| (head) | Control+*pas-5* | 1.467 | 12 (N = 2) | 0.0005 |  |
|  | *skn-1+pas-5* | 0.966 | 9 (N = 2) | 0.460 | 0.0002 |
|  | Control*+rpn-2* | 1.921 | 22 (N = 2) | 1.35E-06 |  |
|  | *skn-1+ rpn-2* | 1.033 | 11 (N = 2) | 0.620 | 2.78E-06 |
