# Supplementary material for: Specific SKN-1/Nrf Stress Responses to Perturbations in Translation Elongation and Proteasome Activity
Source: PLoS Genet. 2011 Jun 9;7(6):e1002119. doi: 10.1371/journal.pgen.1002119 (PMC3111486; doi:10.1371/journal.pgen.1002119)
Supplement: Table S7 — Statistical analysis of in vivo UPS activity experiments. Number of individual experiments is shown in parentheses. Data correspond to results shown in Figure 5 and S4C–S4E. (DOCX) [file pgen.1002119.s013.docx]

**Table S7. Statistical analysis of *in vivo* UPS activity experiments.**

| Strain | Tissue-specific promoter (tissue) | RNAi treatment | Numbers of UbG76V-Dendra2 animals that were imaged | Numbers of Dendra2 animals that were imaged | *P* value against control  (UbG76V-Dendra2) |
| --- | --- | --- | --- | --- | --- |
| N2 | *vha-6* (intestine) | Control | 29 (10) | 8 (6) |  |
|  |  | *skn-1* | 21 (2) | 10 (2) | 3.05E-05 |
|  |  | *eef-1A.1* | 15 (2) | 8 (2) | 0.0003 |
|  |  | *eef-1A.2* | 8 (2) | 5 (1) | 0.0102 |
|  |  | *eef-1B.1* | 9 (2) | 8 (2) | 0.2859 |
|  |  | *eef-1G* | 19 (2) | 11 (2) | 0.02555 |
|  |  | *eef-2* | 9 (2) | 8 (2) | 0.0019 |
|  |  | *ifg-1* | 7 (1) | 7 (1) | 0.2904 |
|  |  | *eif-1* | 10 (1) | 6 (1) | 0.6838 |
|  |  | *eif-1.A* | 10 (1) | 6 (1) | 0.1338 |
|  |  | *pbs-5* | 5 (1) | 7 (1) | 8.32E-05 |
|  |  | *rpn-2* | 13 (1) | 7 (1) | 5.80E-05 |
|  |  | *rpt-4* | 11(1) | 8 (1) | 3.33E-05 |
| N2 | *unc-54* (body-wall muscle) | Control | 16 (2) | 7 (2) |  |
|  |  | *skn-1* | 19 (2) | 10 (2) | 0.1787 |
| *rrf-3 (pk1426)* | *dat-1* (dopaminergic neurons) | Control | 19 (2) | 6 (1) |  |
|  |  | *skn-1* | 20 (2) | 6 (1) | 0.013 |
