# Supplementary material for: Specific SKN-1/Nrf Stress Responses to Perturbations in Translation Elongation and Proteasome Activity
Source: PLoS Genet. 2011 Jun 9;7(6):e1002119. doi: 10.1371/journal.pgen.1002119 (PMC3111486; doi:10.1371/journal.pgen.1002119)
Supplement: Table S8 — Primer sequences. This table includes all primers used in qRT-PCR experiments. (DOCX) [file pgen.1002119.s014.docx]

**Table S8. Primer sequences.**

| Gene name | Forward primer sequence (5’-3’) | Reverse primer sequence (5’-3’) |
| --- | --- | --- |
| *gcs-1* | AATCGATTCCTTTGGAGACC | ATGTTTGCCTCGACAATGTT |
| *atf-5* | CCATCAATCTTATCAACAGCATCAT | CTGGTGGAACCGAAGTG |
| *haf-7* | GACGTGGAAAAGCTGAGAGG | GCAGGGAAAATGTGAGGAAA |
| *gst-4* | CCCATTTTACAAGTCGATGG | CTTCCTCTGCAGTTTTTCCA |
| *gst-10* | GTCTACCACGTTTTGGATGC | ACTTTGTCGGCCTTTCTCTT |
| *F20D6.11* | GGAAATTCTCGGTAGAATCGAA | ACGATCACGAACTTCGAACA |
| *hsp-4* | GAAGCAGATTGCCGAATCC | CATCATTGAAGTATGCTGGCAC |
| *hsp-6* | TCGCCGATGTTCTTCTCGTA | GGGTTGACAGCCTTTGAT |
| *hsp-60* | CAAGGCTCCAGGATTCG | AAAGATCGTTGCTCCCG |
| *T05E11.3* | ATTGTACGTTCGTCGTGT | GTTTGTGCTGTTGGAGGT |
| *daf-21* | CTACTCTACCTCCGCTGG | GCAACAACATCCTTGGACT |
| *dnj-19* | GGTTCTTTCTAGTAAACAAGGAGAT | TTCTGGGTATTTCTTATTTGGCATT |
| *dnj-12* | TGTGCGGATTTGTGCGA | TCGGCATGAGCAATAACCT |
| *rpt-3* | CCCAAGAGGAGTTCTCATGTA | ATGAAGGAAGCAGCAGTATT |
| *rpn-12* | CTGCCAACAGATTGTCCG | GGCGTAGAGATGTAAGCG |
| *pas-4* | CGAGCCATCTGGAGCTTACTA | TCCTCAAGGTATTCACGCAC |
| *pbs-6* | TGGACAGAGCCATCTCATT | CTTCAGCGATGACCAAGTG |
| *skn-1* | GTTCCCAACATCCAACTACG | TGGAGTCTGACCAGTGGATT |
| *act-1* | TCGGTATGGGACAGAAGGAC | CATCCCAGTTGGTGACGATA |
| *tba-1* | ACTGATCTCTGCTGACAAGG | TTGGCTGGCTCGAAGCAA |
