# Supplementary material for: Specific SKN-1/Nrf Stress Responses to Perturbations in Translation Elongation and Proteasome Activity
Source: PLoS Genet. 2011 Jun 9;7(6):e1002119. doi: 10.1371/journal.pgen.1002119 (PMC3111486; doi:10.1371/journal.pgen.1002119)
Supplement: Text S1 — Supplementary Materials and Methods. (DOC) [file pgen.1002119.s015.doc]

**Text S1. Supplementary Materials and Methods**

***C. elegans* strains and transgenesis**

The following strains were used in this study: N2 Bristol (wild type), LD001 *Is007[SKN-1 B/C::GFP; rol-6]* [1], LD1171 *Is003[gcs-1p::GFP; rol-6]* [2], LD1000 *Ex005[gcs-1pΔ2mut3::GFP; rol-6]*, LD1000 *Ex005[gcs-1pΔ2mut3::GFP; rol-6]* [1,2], *sek-1(km4); Ex003[gcs-1p::GFP; rol-6]* [3], CL2166 *dvIs19[pAF15(gst-4p::GFP::NLS)],* CL2070 *dvIs70[pCL25(hsp-16.2p::GFP)]* [4], AM446 *rmIs223[hsp-70p::GFP; rol-6]*, SJ4005 *zcIs4[hsp-4p::GFP]*, EU31 *skn-1(zu135)*, KU4 *[sek-1(km-4)]*, VC390 *[nsy-1(ok593)],* TJ356 *Is[DAF-16::GFP]* and CF1553 *muIs84[pAD76(sod-3::GFP)]*.

For the proteasome activity assay, the promoters *Punc-54*, *Pdat-1*, and *Pvha-6* provided expression of Dendra2 or UbG76V::Dendra2 in the body-wall muscle, dopaminergic neurons, or intestine, respectively [5]. Transgene plasmids were injected into N2 or *rrf-3(pk1426)* animals at 100ng/ul to generate the following strains: YD1 *xzEx1[Punc-54::Dendra2],* YD3 *xzEx3[Punc-54::UbG76V::Dendra2]*, YD21 *rrf-3(pk1426);xzEx21[Pdat-1::Dendra2]*, YD23 *rrf-3(pk1426);xzEx23[Pdat-1::UbG76V::Dendra2]*, YD25 *xzEx25[Pvha-6::Dendra2]*, and YD27 *xzEx27[Pvha-6::UbG76V::Dendra2]*.

**Reporters for proteasome subunit gene expression were generated by fusing either putative promoter or entire coding regions to GFP. Transcriptional (promoter) fusions were created for *pas-5, pbs-4, rpn-2, rpn-11 and rpt-5* by using PCR to amplify 1.2~2kb intergenic promoter sequences together with the first few amino acids of each coding region, then introducing this sequence into the GFP vector pPD118.25** (Andrew Fire Lab Vector Kit, L3786, Addgene) **in place of the *let-858* promoter. The RPN-11::GFP translational fusion construct includes a PCR-amplified 3169-bp sequence covering the 2057-bp promoter region, an 1105-bp coding region with introns, and the 155-bp 3’ UTR of *rpn-11* (substituted for the *let858* 3’ UTR in the vector). To create transgenic GFP reporter strains, 120–180 ng/µl transgene DNA was injected into N2 animals along with 20 ng/µl *myo-2p*::CFP to generate extrachromosomal transgenic lines.** Unless otherwise indicated, worms were cultured at 20ºC on NGM plates that were seeded with a lawn of OP50.

**Brood size assays**

To allow offspring to be counted, L3/L4 animals were transferred individually to a 35mm RNAi plate and allowed to lay eggs, then moved to a fresh RNAi plate every 24 hours egg until egg laying ceased. 14-15 worms were analyzed for each RNAi treatment.

**In-gel proteasome activity assay**

*rrf-3(pk1426)* worms were placed on RNAi plates as L2/L3 larvae, then collected and frozen at -80 ^o^C two days later. Worms were lysed in native gel lysis buffer using a Dounce homogenizer, then native gel electrophoresis and developing of the gel was performed essentially as described previously [6]. For each RNAi or control sample, 40 μg total protein was loaded onto the gel. Gels were run at 40 mA for 3 hours at 4 ^o^C in an ice bath, then the gel was developed in developing buffer with 80 μM of the suc-LLVY-AMC substrate (Bachem), which measures the chymotrypsin-like activity of the proteasome. Fluorescent signal levels were normalized to Coomassie staining, which was performed using the Colloidal Blue Staining Kit (Invitrogen). Signal levels were adjusted using Photoshop 9.0 (Adobe), and analyzed using Fiji.

**Sensitivity to proteasome inhibition**

To assess sensitivity to the proteasome inhibitor MG132, wild type animals that had been exposed to control or *skn-1* RNAi bacteria for three days (since 1-day adulthood) were gathered, washed in M9, and placed in 100µl wells in a 96 well plate at 20^0^C at approximately 50 worms/well. Each well contained 0-10 µM MG132 in 1% DMSO in M9, along with bacterial food supplement. Survival was scored after 24 hours by prodding with a pick.

**References**

1. An JH, Blackwell TK (2003) SKN-1 links C. elegans mesendodermal specification to a conserved oxidative stress response. Genes Dev 17: 1882-1893.

2. Wang J, Robida-Stubbs S, Tullet JM, Rual JF, Vidal M, et al. (2010) RNAi screening implicates a SKN-1-dependent transcriptional response in stress resistance and longevity deriving from translation inhibition. PLoS Genet 6.

3. Inoue H, Hisamoto N, An JH, Oliveira RP, Nishida E, et al. (2005) The C. elegans p38 MAPK pathway regulates nuclear localization of the transcription factor SKN-1 in oxidative stress response. Genes Dev 19: 2278-2283.

4. Link CD, Cypser JR, Johnson CJ, Johnson TE (1999) Direct observation of stress response in Caenorhabditis elegans using a reporter transgene. Cell Stress Chaperones 4: 235-242.

5. Hamer G, Matilainen O, Holmberg CI (2010) A photoconvertible reporter of the ubiquitin-proteasome system in vivo. Nat Methods 7: 473-478.

6. Elsasser S, Schmidt M, Finley D (2005) Characterization of the proteasome using native gel electrophoresis. Methods Enzymol 398: 353-363.
